# Dietary seaweed (*Saccharina latissima*) supplementation in pigs induces localized immunomodulatory effects and minor gut microbiota changes during intestinal helminth infection

**DOI:** 10.1038/s41598-023-49082-5

**Published:** 2023-12-11

**Authors:** Charlotte Smith Bonde, Helena Mejer, Laura J. Myhill, Ling Zhu, Penille Jensen, Nilay Büdeyri Gökgöz, Lukasz Krych, Dennis Sandris Nielsen, Kerstin Skovgaard, Stig Milan Thamsborg, Andrew R. Williams

**Affiliations:** 1 Department of Veterinary and Animal Sciences, University of Copenhagen, Frederiksberg, Denmark; 2 Department of Food Science, University of Copenhagen, Frederiksberg, Denmark; 3 Department of Biotechnology and Biomedicine, Section for Protein Science and Biotherapeutics, Technical University of Denmark, Kongens Lyngby, Denmark

**Keywords:** Parasitic infection, Parasitology, Microbiome, Parasitic infection

## Abstract

Brown seaweeds have a rich bioactive content known to modulate biological processes, including the mucosal immune response and microbiota function, and may therefore have the potential to control enteric pathogens. Here, we tested if dietary seaweed (*Saccharina latissima*) supplementation could modulate pig gut health with a specific focus on parasitic helminth burdens, gut microbiota composition, and host immune response during a five week feeding period in pigs co-infected with the helminths *Ascaris suum* and *Oesophagostomum dentatum*. We found that inclusion of fermented *S. latissima* (Fer-SL) at 8% of the diet increased gut microbiota α-diversity with higher relative abundances of *Firmicutes, Tenericutes, Verrucomicrobia, Spirochaetes* and *Elusimicrobia*, and lower abundance of *Prevotella copri*. In the absence of helminth infection, transcription of immune-related genes in the intestine was only moderately influenced by dietary seaweed. However, Fer-SL modulated the transcriptional response to infection in a site-specific manner in the gut, with an attenuation of infection-induced gene expression in the jejunum and an amplification of gene expression in the colon. Effects on systemic immune parameters (e.g. blood lymphocyte populations) were limited, indicating the effects of Fer-SL were mainly localized to the intestinal tissues. Despite previously documented in vitro anti-parasitic activity against pig helminths, Fer-SL inclusion did not significantly affect parasite egg excretion or worm establishment. Collectively, our results show that although Fer-SL inclusion did not reduce parasite burdens, it may modify the gut environment during enteric parasite infection, which encourages continued investigations into the use of seaweeds or related products as novel tools to improve gut health.

## Introduction

Brown seaweeds are marine macroalgae with bioactive-rich content such as the polysaccharides fucoidan and laminarin (up to > 10% dry matter in some species)^1^. Brown seaweeds (typically at a dietary inclusion rate of 1–2%) and their compounds may provide a range of health benefits for pigs, such as prebiotic and immune-enhancing effects, increased average daily gain, improved digestibility, and stimulation of beneficial bacteria in the gut^2–4^. Effects of dietary brown seaweeds or their extracts on the porcine gut microbiota (GM) vary depending on the brown seaweed type, seaweed extracts, and the use of extracts alone or in combination. Brown seaweeds can modulate the GM by increasing the abundance of *Lactobacillus* spp. and reducing *E. coli*^5,6^*.* Likewise, a diet containing the brown seaweed compounds laminarin and fucoidan alone reduces *Enterobacterium spp.* and increases *Lactobacilli* spp. in pigs, respectively, yet these effects were not apparent when laminarin and fucoidan were used in combination^7,8^. However, it has also been shown that dietary inclusion (0.15% of dietary intake), of two different brown seaweed extracts containing both laminarin and fucoidan reduce enterobacteria, bifidobacteria or lactobacilli populations in pigs regardless of their use alone or in combination^9^. In addition to modulating the GM, dietary seaweed supplementation has also been reported to exert modulatory activity on the host immune system, for example by increasing IgG production, upregulating intestinal mucin, *MUC2* transcription, and lowering pro-inflammatory gene expression^7,10,11^.

Helminth infections are common in livestock, and the interaction between helminths and GM can be critical for the health of the host^12^. There appears to be a complex relationship between helminth infection, GM, and the immune system, but how such interactions can be modulated by bioactive dietary compounds remains unclear in many cases^13^. Moreover, whether manipulation of the gut environment by dietary seaweed inclusion can be a novel treatment for helminth infections is unclear, and is of increasing importance as resistance to conventional anthelmintic drugs is widespread^14^.

In the last two decades, anthelmintic resistance in the porcine nodular worm (*Oesophagostomum* spp.) against all the major drug classes has developed in Europe^15–19^. For this reason, a number of alternative helminth control options such as probiotics or bioactive forages have been investigated in pigs, with the inclusion of plants such as chicory in the diet a promising tool to lower worm burdens^20,21^. In ruminants, where anthelmintic resistance is even more prevalent, many bioactive forages with anti-parasitic properties such as chicory, sainfoin, and birdsfoot trefoil have been demonstrated as a viable alternative option for control of helminth infections^22,23^.

Fermentation technology may be applied to increase both the bioactive properties of plants and plant medicines^24,25^ and the nutrient digestibility of feed^26,27^. Recently, we demonstrated that extracts from fermented and non-fermented brown seaweeds, *Saccharina latissima* and *Laminaria digitata* had potent in vitro activity against *Ascaris suum* infective larvae (L3)^28^. These findings encouraged the present in vivo trials to determine whether inclusion of fermented seaweed (*S. latissima*) in the diet could reduce parasite burdens in pigs. Moreover, given the helminth-GM-immune system interaction and the anti-inflammatory and prebiotic properties of (brown) seaweed, we reasoned that seaweed supplementation may also alleviate infection-associated pathology and positively influence gut health. Thus, we investigated the effect of fermented *S. latissima* in pigs experimentally infected with *A. suum* and *O. dentatum* on possible anti-parasitic effects, alterations in GM composition, and modulatory effects on the immune system.

## Experimental section

### Feed

The seaweed *S. latissima* was provided by Nordic Seaweed Aps (NS), Grena, Denmark. It was harvested in Fjekkefjord Norway 2019 (Lot: ASNT210119) and fermented aerobically as previously described^28^. The same lot was used in the two studies described below, but the batches were fermented, frozen and dried four months apart. The inclusion of seaweed was a 1:1 substitution of 5% (w/w) of the basal diet in Study 1 of fermented (Fer-SL) and non-fermented *S. latissima* (Non-Fer-SL). In Study 2, the seaweed substitution was 8% (w/w) Fer-SL, and the total feed allocated to Fer-SL groups were adjusted to the metabolic energy of the groups fed control diet (Fer-SL groups were allocated 1% more feed, Table 1). Feeding was provided twice daily according to the norm for Danish pig production^29^ for both Study 1 and 2, and water was provided ad libitum.

**Table 1:** Contents and composition of control diet (Study 1 and 2) and 8% dry matter (DM) dried fermented *Saccharina latissima* (Fer-SL) supplemented diet (Study 2). In Study 1, the inclusion of fermented (Fer-SL) and non-fermented *S. latissima* (Non-Fer-SL) was a 1:1 substitution of 5% (w/w) of the control diet.

Ingredient (g/kg)	Study 1	Study 2	
	Control diet	Control diet	Seaweed diet (Fer-SL)*
Barley	561.0	561.0	516.1
Wheat	177.7	177.7	163.5
Soybean meal	203.3	203.3	187.0
Beet molasses	15.0	15.0	13.8
Seaweed meal		–	80
Palm oil distillate fatty acids	15.0	15.0	13.8
Calcium carbonate	13.1	13.1	12.1
Calcium phosphate	5.9	5.9	5.4
Sodium chloride	3.6	3.6	3.3
L-lysine hydrochloride	2.4	2.4	2.2
Methionine	1.0	1.0	1.0
Vitamin/Mineral mix**	2.0	2.0	1.8
Analyzed composition***			
Dry matter (%)	–	87.0	87.2
Ash (% of DM)	–	5.1	5.9
Fat (% of DM)	–	3.4	3.6
Phosphorous (% of DM)	–	–	0.49
Crude protein (% of DM)	–	17.3	17.2
Metabolizable energy (MJ/kg)****	–	13.5	13.4

*Ingredients (g/kg) calculated as 92% of the control feed.

**per kg of feed: Vitamin A (4200 i.e.); Vitamin D3 (420 i.e.); E-Vitamin/DL-tocopheryl actetae (84 i.e.); Vitamin B1 (2.1 mg); Vitamin B2 (2.1 mg); Vitamin B6 (3.2 mg); Vitamin B12 (0.02 mg); Calcium-D-pantothenate (11 mg); Nicotinic acid (21 mg); Biotin (0.05 mg); Vitamin K3 (2.1 mg); Iron sulphate (84 mg); Copper sulphate (15 mg); Manganese oxide (42 mg); Zinc oxide (100 mg); Calcium iodate (0.21 mg); Sodium selenite (0.3 mg).

***Analyzed composition was done on prepared feed for both diets in Study 2.

****Calculated based on Danish feed units (FE).

### Parasites

The batch of *O. dentatum* L3 (EH-strain) used for both studies was produced from larval faecal cultures and kept in tap water at 10 °C until use^30^. The *A. suum* eggs were isolated and embryonated as described previously^28^.

### Animals

Study 1: Study 1 was designed as a preliminary investigation to assess palatability and potential indications of anti-parasitic activity. Nine castrated male pigs [Duroc x (Yorkshire x Landrace)] were obtained from a Danish certified specific pathogen-free (SPF) farm with no previous history of helminth infection. The pigs were ∼9 weeks of age and weighed 25.6 ± 2.5 kg (mean ± standard deviation (SD)) at arrival. All animals were helminth-free as confirmed by absence of helminth eggs in faeces and anti-*Ascaris* antibodies in serum. During the study, pigs were housed on a group-basis in solid concrete-floored pens with wood shavings. Although we acknowledge that co-housing may not be optimal for assessing gut microbiota changes, individual housing was not possible for practical and ethical reasons. Animal welfare checks were performed daily, and body weight and faecal consistency (scored 1–5 where 3 or above is diarrhoea) were recorded weekly. Separate tools and boots were used in each pen to prevent cross-contamination between groups.

Study 2: This study was designed as larger study to assess in more detail anti-parasitic effects as well as dietary effects on parasite-induced immunity and GM modulation. Thirty-two crossbred [Duroc x (Yorkshire x Landrace)] pigs (16 castrated males, 16 females) were purchased from the same provider listed above. The pigs were ∼9 weeks of age and weighed 26.0 ± 1.7 kg (mean ± SD) on arrival. The animals were housed and managed as in Study 1. All pigs were vaccinated against *Lawsonia intracellularis* with one dose of a live, attenuated vaccine (Enterisol® Ileitis, Boehringer Ingelheim, DK) on-farm 40 days prior to the first infection and confirmed helminth free by absence of eggs in faeces counts at arrival and serology for *A. suum* and *O. dentatum* by ELISA.

### Experimental design

Study 1: The experiment was set up as a parallel study of three diets. After stratification for body weight, the pigs were randomly allocated into three groups (n = 3), housed in 5 m^2^ pens, with groups fed either control feed (C), control feed added Non-Fer-SL (NSL), or control feed added Fer-SL (FSL). All pigs were provided with their respective diets from arrival. Six days after arrival all animals were inoculated with 6000 *O. dentatum* third stage larvae (L3) (0 days post infection (dpi)), and 8 days later infected with 2000 *A. suum* infective eggs (8 dpi) by stomach tube. Twenty-two days post infection with *O. dentatum* (22 dpi), pigs were euthanized by captive bolt, followed by exsanguination and evisceration (Supplementary Figure 1).

Study 2: This was a 2 × 2 factorial study with diet and infection. After stratification for sex and body weight, the thirty-two pigs were randomly allocated into four groups (housed in 5 m^2^ pens): UC (uninfected pigs fed control diet), IC (infected pigs fed control diet), USL (uninfected pigs fed Fer-SL diet) and ISL (infected pigs fed Fer-SL diet). The diets were offered from arrival, and groups IC and ISL were one week later inoculated with 6000 *O. dentatum* L3 (0 dpi) and two weeks later with 2000 *A. suum* infective eggs (14 dpi), using a stomach tube. Twenty-eight days post infection with *O. dentatum* (28 dpi), pigs were euthanized (Supplementary Figure 2) as mentioned above. 14 days of *A, suum* infection was judged optimal to measure larval counts before the onset of larval expulsion which occurs around 17–21 days dpi^31^ as well as being a time point where strong immune responses and changes in GM composition have been detected in the gut tissues as result of infection^32–34^.

### Parasitological techniques

Rectal faecal samples were collected weekly and examined for faecal nematode egg counts (FEC) of by a modified concentration McMaster technique, with an analytical sensitivity of 20 eggs per gram faeces (EPG)^30^. Additional samples were collected in Study 1 at 19–22 dpi, and for Study 2 at 17, 21, 24 and 28 dpi for FEC. In Study 2, hatching of *O. dentatum* eggs and larval development were quantified for faecal samples at 24 dpi. Faecal larval cultures of 5 g faeces were set up in triplicates for each infected animal and the L3 were harvested 18 days later^30^. The larval development percentage was calculated based on the total number of L3 recovered divided by the number of eggs in the faecal culture (i.e. EPG × 5 g).

At necropsy, livers were macroscopically examined for liver spots on the surfaces. The small intestine was rinsed with warm saline and a 50% subsample of the contents and washings embedded in agar (agar powder, product code 20768.292; VWR, Denmark) to isolate minute *A. suum* larvae^35^. The larvae were harvested using a 20 µm sieve after 3 h and preserved in 70% ethanol. The same procedure was applied for the large intestine (caecum and colon together) but only two 5% subsamples were embedded in agar and samples were harvested using a 38 µm sieve after 20 h^36^.

### Immunology samples (Blood, PBMCs, CLN, Flow cytometry)

In both studies, blood samples were collected from pigs by jugular venipuncture using vacutainer serum separator tubes (BD Vacutainer ®, SST^TM^II *Advance*) at arrival, 0 dpi, and at necropsy (22 or 28 dpi, for Study 1 and 2, respectively). After storage for 24 h at 5 °C, serum was separated by centrifugation (10 min at 1580 RCF) and stored at − 80 °C until use. At necropsy (28 dpi) in Study 2, blood was also collected in heparin-coated tubes (BD Vacutainer®) and PBMCs were isolated as described^37^. Cells were isolated from the ileo-caecal lymph nodes (CLN) of the pigs at necropsy. Flow cytometry was performed on both PBMC and CLN cells as described^37^. Ex vivo cell stimulation was performed on PBMCs to assess the production of inflammatory cytokines. Cells were stimulated for 24 h with Lipopolysaccharides (LPS) (1 µg/mL) and the supernatant was collected and frozen. Secreted tumour necrosis factor-α (TNF-α) and interleukin 1β (IL-1β) were quantified using commercial antibody pairs (Duosets; R&D systems; UK). An ELISA on Immunoglobulin G1 (IgG1) (Study 1) or total IgG (Study 2) levels in serum against *A. suum* adult body fluid (ABF) on samples at arrival and on day of necropsy. The ELISA was conducted as described^37^, utilizing mouse anti-pig IgG1 (clone K139 3C8; Bio-Rad, CA, USA) followed by goat anti-mouse IgG:HRP (Bio-Rad), or goat anti-pig IgG:HRP. Study 2 also included an ELISA on total IgG levels in serum against *O. dentatum* excretory/secretory (E/S) products on samples at arrival and on day of necropsy.

### Tissue samples (Histology and Gene expression)

For Study 2 tissue samples were taken from the mid-point of the jejunum and proximal colon (20 cm distal of the ileo-caecal junction). The samples were fixed in 4% formaldehyde, paraffin-embedded, sectioned, and stained with Periodic Acid Schiff stain to evaluate goblet cells and villi/crypt ratios. For determination of goblet cell numbers, 5 random microscopy fields of each tissue section were counted by a single-blinded observer using a calibrated counting grid covering a total area of 0.25 mm^2^ at 200 × magnification. Cell counts were expressed as cells/mm^2^ tissue. For villous/crypt ratios, 5 well-orientated villus/crypt units were randomly selected from each tissue section by a blinded observer, imaged using a Leica DFC480 camera and measurements performed using LAS v4.6 software (Leica, Switzerland). Additional jejunum and colon tissue samples were collected for gene expression analysis. The samples were gently washed in PBS and stored in RNA later at − 20 °C until use. RNA was extracted from the samples using a miRNeasy Kit (Qiagen, Denmark) ^37^. The RNA concentration and purity were measured on a Nanodrop, RNA integrity was measured on a bioanalyzer and stored at − 80 °C until cDNA synthesis. cDNA synthesis of 500 ng total RNA was carried out using Qiagen QuantiTect Reverse Transcription Kit following the manufacturer’s protocol. The expression levels of a panel of genes of interest and reference genes were examined by microfluidic qPCR (BioMark, Fluidigm, San Francisco, CA, USA) after 19 cycles of preamplification^37^. Microfluidic qPCR was performed in a 96.96 Dynamic Array (Fluidigm), using the following cycle conditions: 2 min at 50 °C and 10 min at 95 °C for thermal mix, followed by 35 cycles with denaturation for 15 s at 95 °C and annealing/elongation for 1 min at 60 °C. After each run, melting curves were generated to confirm a single PCR product (increasing 1 °C/3 s). After data pre-processing 75 genes of interest for proximal colon and 80 genes of interest for jejunum were statistically analyzed. The data pre-processing and normalization to three validated reference genes *B2M*, *PL13A* and *TBP* were carried out as described^38^. The list of genes and primers is presented in Supplementary Table 1.

### Gut microbiota analysis (Digesta sampling, DNA Extraction, 16S rRNA gene amplicon sequencing and data processing)

Fresh intestinal digesta samples were removed from the proximal colon (20 cm distal of ileo-caecal junction) of each pig for GM sampling in Study 2. The samples were snap-frozen in liquid nitrogen and stored at − 80 °C. Additional digesta samples were taken for pH measurement using a Hi2211 pH/ORP Meter (Hanna Instruments). DNA extraction from digesta samples was performed by using the Qiagen QIAmp Fast DNA Stool Mini Kit (Cat. No. 51604), following the manufacturer’s protocol. The extracted DNA was stored at − 20 °C until further analysis. Nanodrop 1000 Spectrophotometer (Thermo Fisher Scientific, USA) and Varioskan Flash (Thermo Fisher Scientific, USA) were used for DNA purity and concentration measurements, respectively.

To determine GM composition, near full-length 16S rRNA gene was amplified with protocol involving multiple forward and reverse primers (Supplementary Table 2). Two-step PCR was performed for the amplification. First PCR conditions were as follows: 95 °C for 5 min, 2 cycles of 95 °C for 20 s, 48 °C for 30 s, 65 °C for 10 s, 72 °C for 45 s, and a final extension at 72 °C for 4 min. A second PCR step was done to barcode the first PCR products with the following conditions: 95 °C for 2 min followed by 33 cycles of 95 °C for 20 s, 55 °C for 20 s, 72 °C for 40 s, and a final extension at 72 °C for 4 min. PCR products were cleaned up using SpeedBeads magnetic carboxylate (obtained from Sigma Aldrich) after each PCR step. The size of barcoded PCR products was checked on 1.5% agarose gel.

The Nanopore sequencing library was constructed according to the ligation sequencing kit SQK-LSK109 protocol. The library was loaded on R9.4 flowcell and sequenced with GridIONX5 platform (Oxford Nanopore Technologies, Oxford, UK). Sequencing data was collected using Nanopore sequencing software GridION version 21.02.5 (https://nanoporetech.com). Guppy version 4.5.2 (https://nanoporetech.com) was used for base calling and demultiplexing. Next, filtering and trimming of demultiplexed sequences (min = 1300 bp, max = 1600 bp, q score ≥ 10) were performed by Nanofilt version 2.7.1^39^. Taxonomy assignment was done by parallel_assign_taxonomy_uclust.py script of Quantitative Insights into Microbial Ecology (Qiime) 1 version 1.8.0^40^. Greengenes database version 13.8^41^ was used as a reference database. The reads classifications did not contain UMI correction because of the low coverage of UMI clusters.

### Statistical analysis

GraphPad Prism 7 (GraphPad Software Inc., CA, USA) or R (version R i386 3.5.2) was used for statistical analysis. Normally distribution of the data was tested using Shapiro–Wilk’s normality test. Normally distributed data was tested using one-way ANOVA or T-test (Study 1) or 2-way ANOVA or T-test (Study 2). Non-normally distributed data was analysed using Mann–Whitney test. FEC area under the curve (AUC) was calculated in Prism using the formula ΔX*([(Y1 + Y2)/2]-Baseline]) where ΔX refers to the time period being assessed, Y1 and Y2 are the egg counts at the start and end of the specified period, and baseline is the minimum FEC value in the area being assessed (0 in this case). An individual value was obtained for each pig, and groups compared using t-test. Statistical significance was assigned at P ≤ 0.05.

For bioinformatics of 16S rRNA gene amplicon sequencing data, Qiime 2 version 2020.6.0^42^ was used to rarefy data to 15,000 reads per sample, and rarefied data were then processed in RStudio version 1.3.1073^43^ using R version 4.0.2^44^ and R packages phyloseq^45^, tidyverse^46^, ggpubr^47^, and reshape2^48^. Alpha diversity was evaluated based on observed features and Shannon index. Principal coordinate analysis (PCoA) plots were generated based on Bray–Curtis and Jaccard distances dissimilarity metrics. Analysis of differentially abundant bacteria was performed by using the DESeq2 package^49^ and DESeq2-based heatmaps showing the significantly differed bacteria (adjusted *p* value < 0.05) were plotted using pheatmap^50^ and RColorbrewer^51^ packages. Pairwise Wilcoxon rank-sum test with Benjamini–Hochberg method from R stats package^44^ was used to obtain adjusted P-values for alpha diversity measures. For beta diversity, adjusted P-values were calculated by pairwise comparisons using permutation MANOVAs on a distance matrix with Holm method from R package RVAideMemoire^52^.

## Results

### Clinical observations

No clinical signs of infections were observed in the pigs, except for a mild cough around 7–9 days after *A. suum* infection, which is associated with migration of the larvae to the lungs. There were no significant differences in daily weight gains between groups in either Study 1 when pigs were fed a 5% inclusion or Study 2 when fed an 8% inclusion (Tables 2, 3). One pig in the IC group in Study 2 had mild diarrhoea at 14 dpi.

**Table 2:** Parasitological data and daily weight gain (DWG) for pigs (n = 3) in Study 1 for control group (C), fermented seaweed inclusion group (FSL) and non-fermented seaweed inclusion group (NSL). Optical density (OD) values on serum levels of IgG1 against *Ascaris suum* adult body fluid (ABF) 22 days post infection*.* Area under the curve (AUC) for *Oesophagostomum dentatum* faecal egg counts (FEC), based on individual eggs per gram faeces (EPG) days 19, 20, 21 and 22 post infection. The data are presented as mean ± S.D.

	C	NSL	FSL	*P* value*	
				NSL	FSL
Worm counts					
* O. dentatum*	6373 ± 455	6170 ± 1011	6700 ± 389	0.77	0.40
* A. suum*	877 ± 381	1025 ± 247	1061 ± 252	0.60	0.52
Liver spots	335 ± 85	289 ± 65	342 ± 94	0.49	0.93
OD ELISA IgG1 (22 dpi)	0.07 ± 0.12	0.05 ± 0.04	0.23 ± 0.10	0.82	0.25
FEC AUC	24,807 ± 5,009	14,387 ± 2,214	13,400 ± 4392	0.12	0.15
DWG (kg)	0.78 ± 0.14	0.75 ± 0.12	0.76 ± 0.05	> 0.99	0.60

*P value in relation to C.

### Seaweed does not significantly reduce parasite burdens

**Figure 1:**
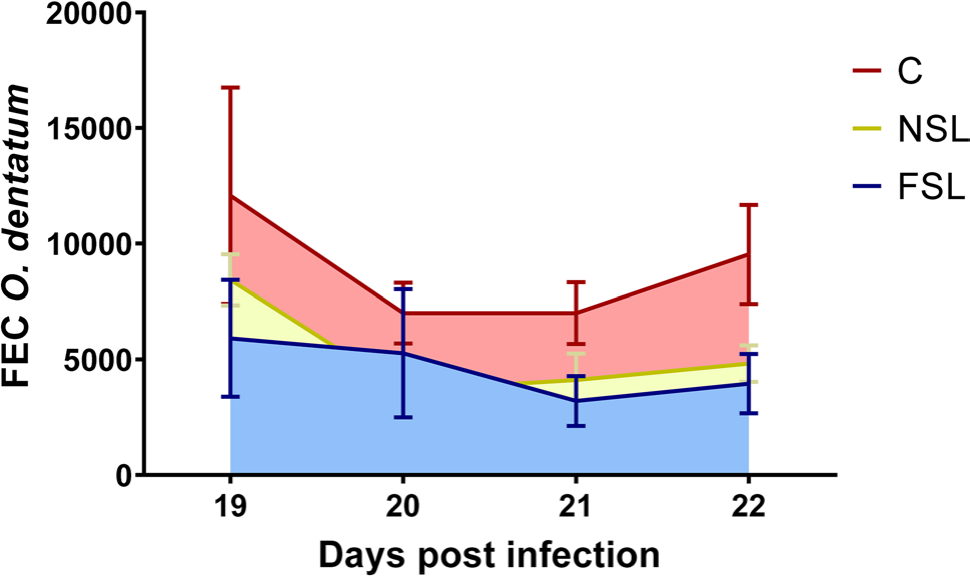
Area under the curve (AUC) of faecal egg counts (FEC), based on eggs per gram faeces (EPG) of individual pigs on days 19, 20, 21 and 22 post infection with *Oesophagostomum dentatum* in Study 1 (n = 3) for control group (C), non-fermented seaweed inclusion group (NSL) and fermented seaweed inclusion group (FSL). Data are presented as mean ± SEM.

Study 1: *A. suum* and *O. dentatum* worms were recovered from all animals, with no significant differences between groups (Table 2). However, *O. dentatum* FEC based on individual EPG at 19 to 22 dpi showed a decrease of 46.0% and 42.0% for FSL and NSL, respectively, as compared to the controls (Fig. 1). However, this was not significant, either by comparing the control group with the two individual seaweed groups (*p* = 0.12 and 0.15 by AUC analysis for NSL and FSL, respectively; Table 2), or by pooling the seaweed groups and comparing to the control (*p* = 0.09 by unpaired t-test).

Study 2: *A. suum* and *O. dentatum* worms were recovered from all infected animals while no worms or liver spots were found in uninfected animals. Infected pigs had significantly higher *Ascaris*-specific and *Oesophagostomum*-specific IgG levels in serum than uninfected pigs within the two feeding groups (*P* < 0.01), but no difference was found in specific IgG levels between the ISL group and the IC group for either *A. suum* or *O. dentatum*. No significant differences were found in any of the parasitological/pathological parameters between the IC and the ISL group (Table 3).

**Table 3:** Parasite data and daily weight gain (DWG) in Study 2 control fed pigs with (IC) or without (UC) parasite infection (*Oesphagostomum dentatum* and *Ascaris suum*) or pigs fed fermented *Saccharina latissima* (Fer-SL) with (ISL) or without (USL) parasite infection (n = 8). Optical density (OD) values on serum levels of IgG against *A. suum* adult body fluid (ABF) and *O. dentatum* Excretory/Secretory (E/S) products 28 days post infection*.* Area under the curve (AUC) for *O. dentatum* faecal egg counts (FEC), based on individual eggs per gram faeces (EPG) days 17, 21, 24 and 28 post infection. The data are presented as mean ± S.D.

	Control diet		Fer-SL diet		*P* value**
	UC	IC	USL	ISL	IC versus ISL
Worm counts					
* O. dentatum*	0	4651 ± 1708	0	5590 ± 776	0.08
A. suum*	0	1189 ± 269*	0	1241 ± 420*	0.78
*O. dentatum* female/male ratio	n.a	1.02 ± 0.13	n.a	0.99 ± 0.14	0.67
FEC AUC	0	44,808 ± 12,889	0	48,853 ± 14,670	0.84
L3 *O. dentatum* development	n.a	41% ± 18%	n.a	29% ± 8%	0.11
Liver spots	0	262 ± 74.85	0	200 ± 83.41	0.15
OD ELISA IgG (28 dpi)					
* O. dentatum*	0.09 ± 0.05	0.57 ± 0.24	0.14 ± 0.15	0.39 ± 0.10	0.13
* A. suum*	0.22 ± 0.05	0.64 ± 0.26	0.21 ± 0.07	0.57 ± 0.0.38	0.65
DWG (kg)	0.76 ± 0.10	0.77 ± 0.13	0.76 ± 0.09	0.75 ± 0.10	0.76

*Based on n = 7 for infection groups.

**P value of IC versus ISL.

*n.a.* not applicable.

### Seaweed changes the gut microbiota composition

To explore the effects of Fer-SL inclusion on the intestinal environment and GM, we first measured pH values of the intestinal content in jejunum and proximal colon. Average pH values were 6.4 (jejunum) and 6 (proximal colon), but no significant effects of either diet or infection were found in either study (data not shown). We next profiled the GM composition in the proximal colon of pigs from Study 2 using Oxford Nanopore based 16S rRNA gene amplicon sequencing.

**Figure 2:**
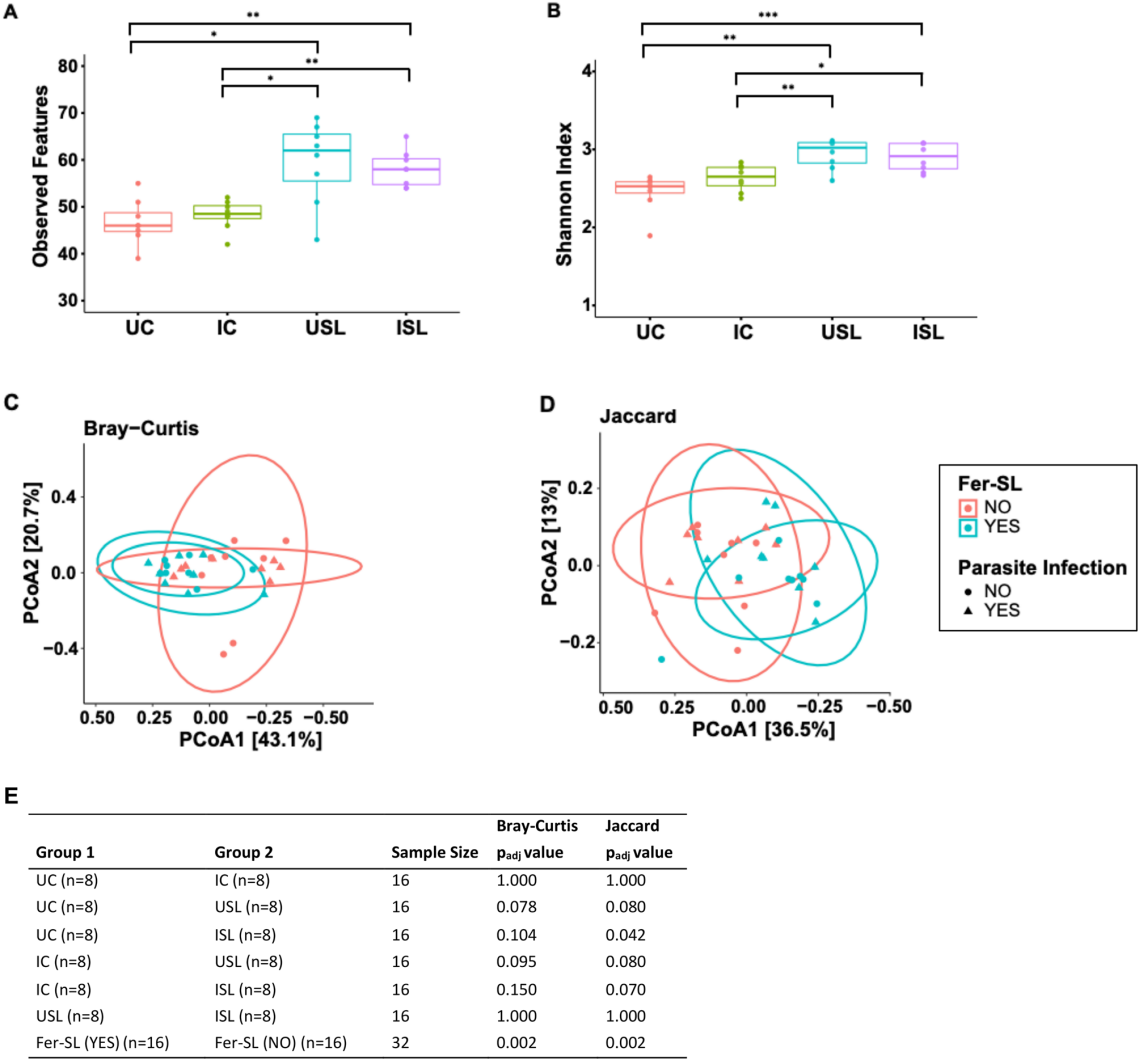
Gut microbiota diversity of pigs in Study 2. Upper panel: Box plot of α-diversity based on (**A**) observed features and (**B**) Shannon index of gut microbiota from proximal colon of groups of pigs (n = 8) in Study 2: control group (UC), fermented *Saccharina latissima* supplementation (USL), *Oesphagostomum dentatum* and *Ascaris suum* infection (IC), or *O. dentatum* and *A. suum* infection combined with *S. latissima* supplementation (ISL). The horizontal line in each box shows the median value. The lower and upper boundaries of boxes are the 25th and 75th quartiles, respectively. The significance of difference was assessed by pairwise Wilcoxon rank-sum test with Benjamini–Hochberg method (*P < 0.05, ** *P* < 0.01, ***P < 0.001). Lower panel: Principal coordinates analysis (PCoA) plots based on Bray–Curtis (**C**) and Jaccard (**D**) distances for beta diversity. Each point on PCoA plots shows the sample, which was coloured and shaped by Fer-SL diet and parasite infection, respectively. The percentage in parenthesis is the percentage variation captured by first and second PCoA axes. Ellipses represent 95% confidence interval. (**E**) Statistical pairwise group comparisons for beta diversity using permutation MANOVAs on a distance matrix with Holm adjustment method. *P* value < 0.05 was considered as significant.

We observed increases in GM alpha diversity as a result of feeding seaweed, both with observed features and Shannon Index, whereas infection had no significant effect (Fig. 2A, B). Beta diversity analysis based on Bray–Curtis dissimilarity (Fig. 2C) and Jaccard (Fig. 2D) indices showed no clear differences between GM composition between uninfected (UC and USL) and infected (IC and ISL) groups (*P* > 0.05), respectively. However, there was a marked effect of Fer-SL inclusion on both beta-diversity metrics (Fig. 2C, D; *P* = 0.002). All pairwise group comparisons results for beta diversity are listed in Fig. 2E. Notably, the effect of seaweed was identical across pens (regardless of parasite infection), suggesting a biological effect of seaweed consumption rather than a pen effect.

**Figure 3:**
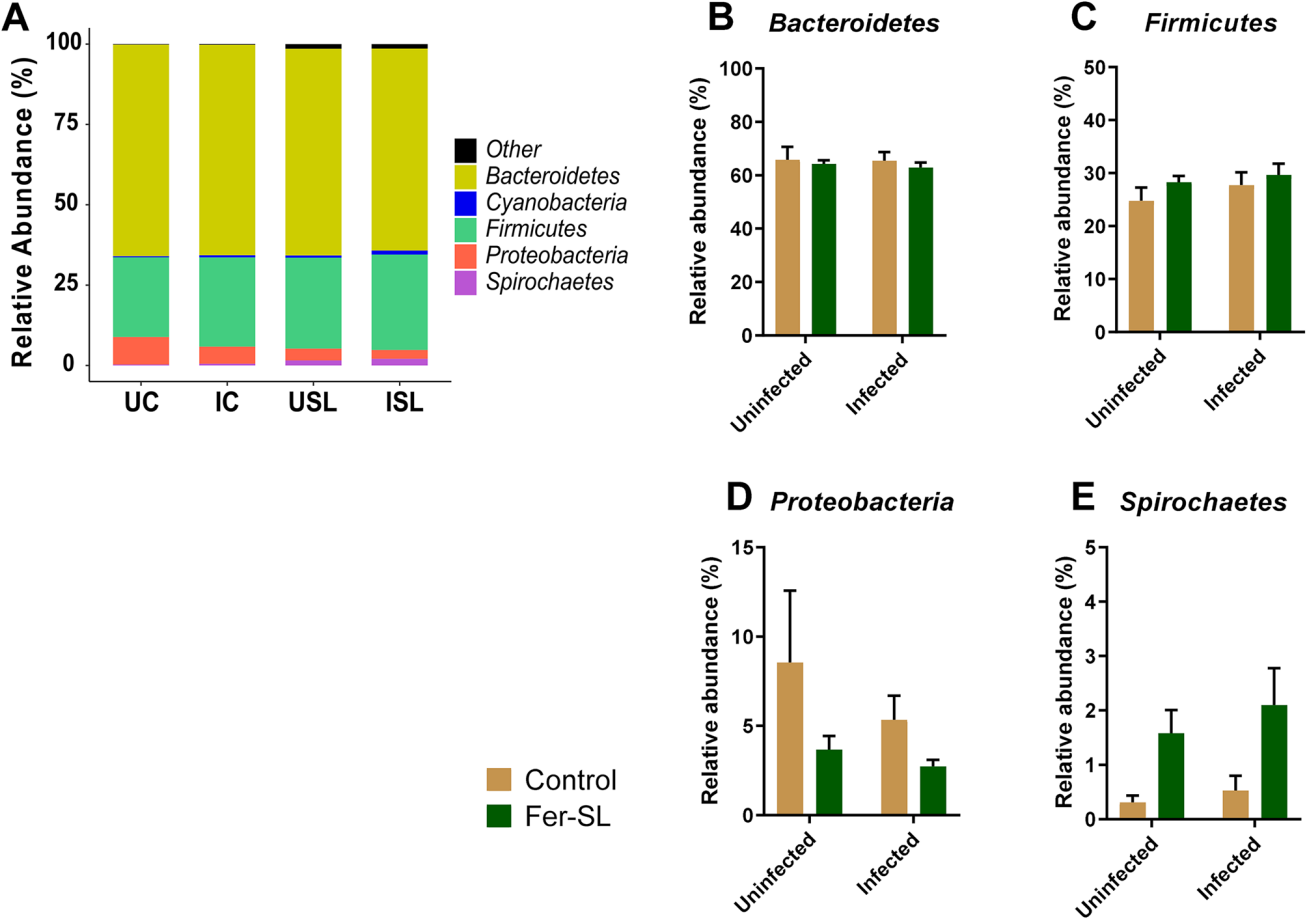
Phylum-level gut microbiota composition of pigs in Study 2. In the left panel, (**A**) Stacked bar plots show relative abundance of phyla in groups of pigs (n = 8) for control fed pigs with (IC) or without (UC) parasite infection (*Oesphagostomum dentatum* and *Ascaris suum*) or pigs fed fermented *Saccharina latissima* (Fer-SL) with (ISL) or without (USL) parasite infection (n = 8). “*Other”* indicates the phyla with median relative abundance below 1%. In the right panel, bar plots show alterations induced by diet and parasite infection for (**B**) *Bacteroidetes*, (**C**) *Firmicutes*, (**D**) *Proteobacteria*, and (**E**) *Spirochaetes*. Data for B-E are presented as mean ± SEM.

**Figure 4:**
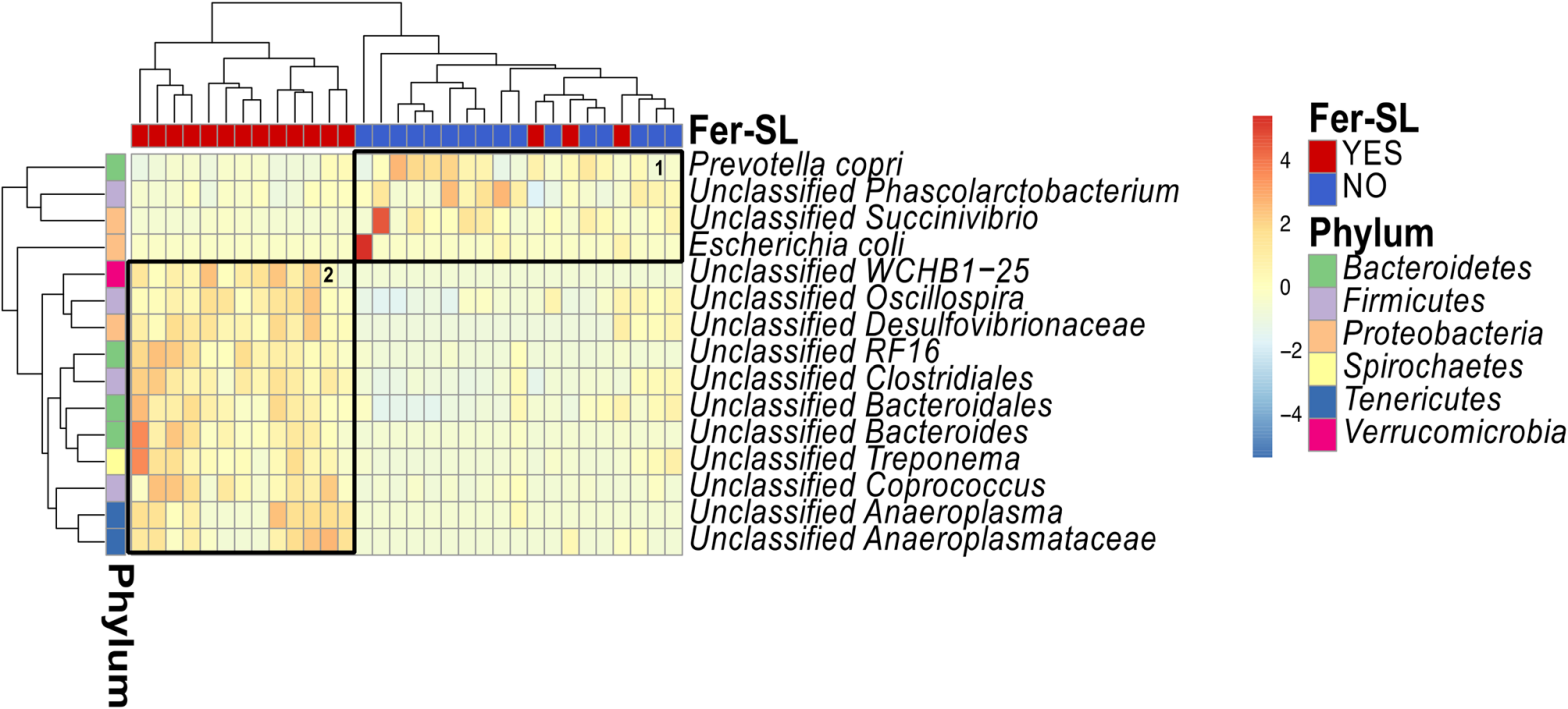
DeSeq2-based heatmap for Study 2. Heatmap shows the significantly differed bacteria at species level in pigs fed with diet with fermented *Saccharina latissima* (Fer-SL) (red) and without Fer-SL diet (blue). Rectangles on heatmap comprise the increased bacteria in groups. Heat map was created using pheatmap, v1.0.12, https://cran.r-project.org/web/packages/pheatmap/index.html^50^.

We next assessed the changes in GM composition at phylum level and found that the GM of all groups was mainly dominated by the *Bacteroidetes* and *Firmicutes* phyla (Fig. 3). To identify bacteria with significantly different abundance between pigs fed the control diet (UC and IC) or Fer-SL diet (USL and ISL), we performed DESeq2-based heatmap analysis of the GM. At phylum level, our findings revealed that Fer-SL diet inclusion resulted in the increased levels of *Elusimicrobia* (*P* = 0.04), *Tenericutes* (*P* < 0.0001), *Spirochaetes* (*P* = 0.02) and *Verrucomicrobia* (*P* = 0.02), but it should be noted that all these taxa were of relatively low abundance. The abundance of *Proteobacteria* tended to be reduced by Fer-SL inclusion in diet (*P* = 0.06). At the species-level, analysis showed a clear grouping as a result of diet (Fig. 4). The pigs fed the control diet had a higher relative abundance of *Prevotella copri* and an unclassified *Phascolarctobacterium*. The GM of pigs fed Fer-SL were characterised by increased relative abundance of *Oscillospira*, *Desulfovibrionaceae*, *RF16*, *Clostridiales*, *Bacteroidales*, *Bacteroides*, *Treponema*, *Coprococcus*, *Anaeroplasma*, and *Anaeroplasmataceae* spp.

Overall, these data indicate that Fer-SL diet inclusion increased alpha diversity of the GM and modulated the GM composition, and that this effect was largely independent of underlying helminth infection.

### Seaweed modulates the immune response to parasite infection

We next investigated how Fer-SL inclusion affected intestinal morphology and expression of genes related to immune responses and epithelial barrier function. We found no significant effect of diet or infection on goblet cell numbers, nor the villi/crypt ratio or crypt depth in either the jejunum or proximal colon in Study 2. However, goblet cell numbers in the jejunum tended to increase in Fer-SL-fed, uninfected animals (USL vs UC, *P* = 0.09), whilst infection in control-fed pigs tended to increase villus/crypt ratios (UC vs IC, *P* = 0.08) in the jejunum (Supplementary Figure 3).

**Figure 5:**
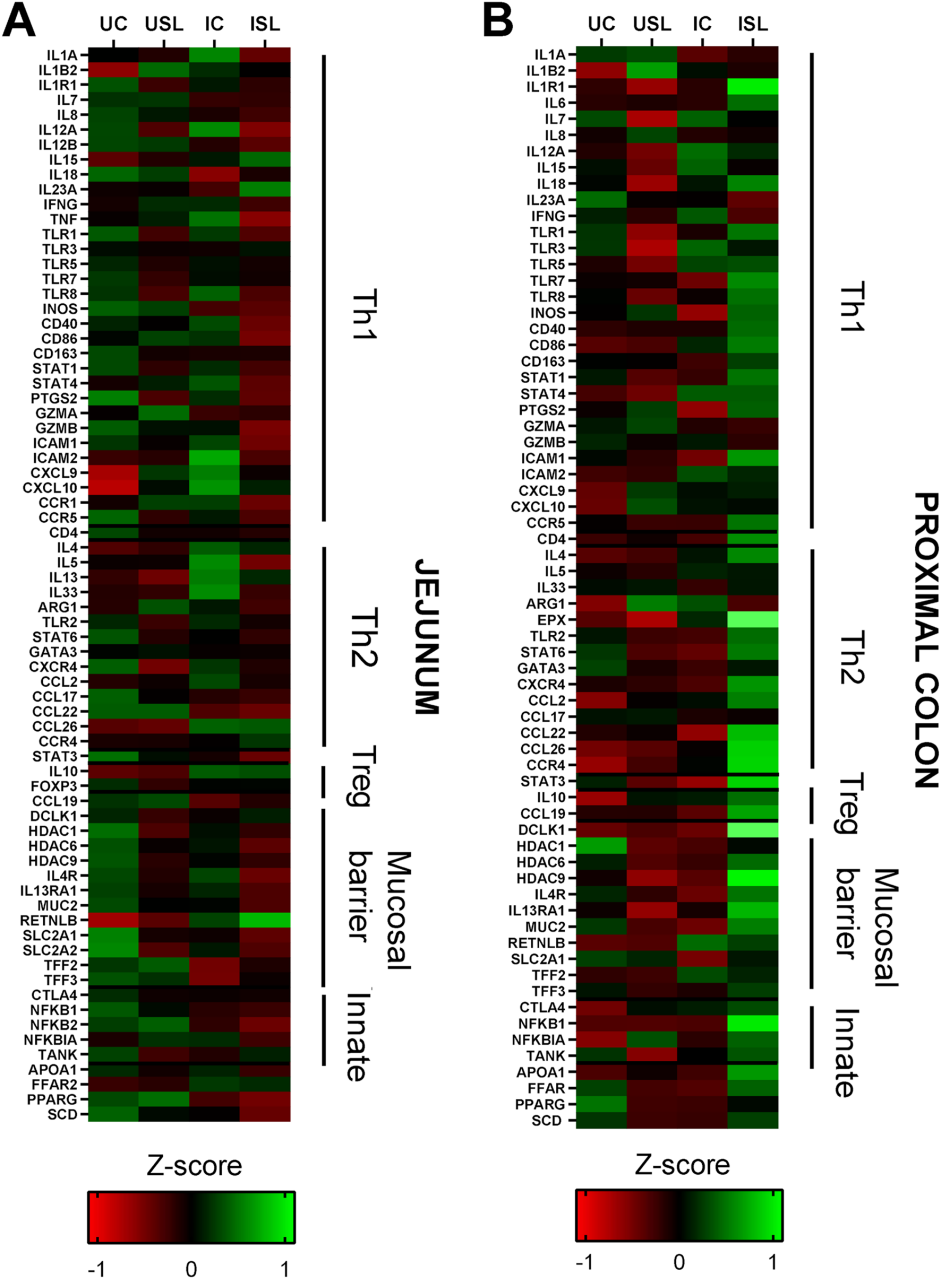
Expression of genes involved in different immunological functions in groups of pigs (n = 8) as a result of *Oesphagostomum dentatum* and *Ascaris suum* infection (IC), fermented *Saccharina latissima* supplementation (USL), or *O. dentatum* and *A. suum* infection combined with fermented *S. latissima* supplementation (ISL). The control group (UC) received no infection or S*. latissima* inclusion. Data presented as Z-scores of relative gene expression data for (**A**) jejunum and (**B**) proximal colon. Heat maps were created using GraphPad Prism 7 (GraphPad Software Inc., CA, USA), https://www.graphpad.com.

To explore whether the dietary inclusion of Fer-SL modulated the intestinal transcriptional response during infection, the expression of a panel of genes covering lipid metabolism, immune and mucosal barrier function was assessed in jejunum and colon tissue. In the jejunum, the diet had only a modest effect on gene expression, but we did note significantly decreased expression of the pro-inflammatory genes *IL12A* and *PTGS2* (Fold change − 1.2 and − 1.5, respectively) (Supplementary Figure 4)*.* Infection resulted in increased expression of immune-related genes in the jejunum, particularly those involved in type-2 responses (Fig. 5). In particular, the expression of *IL13* and *RETNLB* was significantly increased in infected pigs (Supplementary Figure 5), regardless of diet (main effect of infection with a fold change of 3.7 and 1.7, respectively), and the expression of *CCL26*, *IL10* and *TFF2* also tended to be increased (Table 4). Conversely, expression of the pro-inflammatory *IL18* in the jejunum was significantly decreased by infection (fold change − 1.2), and so were type-2 associated gene *CCL22* (fold change − 2.1) and adipocyte regulator *PPARG* (fold change − 1.3). Interestingly, we noted that, across the genes we measured, expression in the jejunum was consistently lower in the ISL group, relative to IC (Fig. 5). This was evidenced by significant, or close to significant, interactions between diet and infection for the expression of *CXCL9, CXCL10*, and *TNF* (Table 4; Supplementary Figure 6), indicating that infection-induced changes in gene expression were attenuated by inclusion of dietary Fer-SL. Thus, although Fer-SL inclusion had limited effects on the gene expression in the jejunum in uninfected pigs, it tended to modulate the response to infection.

**Table 4:** Relative expression and significance (P-value) of genes influenced by diet, infection or their interaction in porcine jejunal tissue from groups of pigs (n = 8): control group (UC), *Oesphagostomum dentatum* and *Ascaris suum* infection (IC), fermented *Saccharina latissima* supplementation (USL), or *O. dentatum* and *A. suum* infection combined with fermented *S. latissima* supplementation (ISL) in Study 2. * indicates *p* ≤ 0.05, # indicates a trend of effect where *p* ≤ 0.1.

Immune function	Immune gene	Relative expression				Significance (*P* value)		
		UC	IC	USL	ISL	Diet	Infection	Interaction
Th1	*IL12A*	4.0	4.6	3.3	2.9	*0.03		
	*IL15*	3.3	3.8	3.4	4.2		0.14	
	*IL18*	2.7	1.9	2.7	2.4		*0.05	
	*TNF*	10.8	18.1	13.3	9.6			#0.08
	*TLR*	6.6	6.5	5.3	4.5	0.14		
	*TLR8*	3.5	4.0	2.7	2.6	#0.09		
	*INOS*	19.9	8.9	13.8	6.6		#0.07	
	*STAT4*	1.8	2.0	1.9	1.6			0.14
	*PTGS2*	4.5	4.1	3.0	2.7	*0.03		
	*ICAM2*	1.6	2.0	1.6	1.6	0.14		0.11
	*CXCL9*	2.7	4.8	4.0	3.5			*0.04
	*CXCL10*	2.3	3.8	3.3	3.2			*0.05
	*CCR1*	6.3	8.8	7.7	4.2			0.13
Th2	*IL4*	2.5	4.1	3.3	3.2		0.14	
	*IL5*	2.0	2.4	2.1	1.7			0.12
	*IL13*	7.6	28.6	6.0	15.1		*0.009	
	*IL33*	2.6	5.3	2.8	2.5			0.12
	*CXCR4*	6.4	6.4	4.5	5.2	0.12		
	*CCL22*	29.4	13.9	21.0	10.0		*0.03	
	*CCL26*	2.9	3.9	2.7	3.8		#0.06	
*Th17*	*STAT3*	5.4	3.3	3.6	2.6		0.15	
T-reg	*IL10*	1.6	2.0	1.6	1.9		#0.06	
	*CCL19*	3.0	1.9	2.9	2.3		0.14	
Mucosal barrier function	*IL4R*	10.4	10.1	8.2	6.5	0.11		
	*RETNLB*	1.5	2.7	1.8	3.6		*0.002	
	*TFF2*	2.4	1.9	2.5	2.1		#0.09	
	*TFF3*	2.6	1.9	2.4	2.3		0.13	
Innate	*NFKB2*	3.7	3.3	4.0	3.0		0.11	
Lipid metabolism	*PPARG*	2.8	2.2	3.2	1.9		*0.03	

In the proximal colon, we also found that diet only had modest effects, with a decrease in the expression of the pro-inflammatory cytokine-encoding gene *IL7* (fold change − 1.2), as well as increases in genes encoding innate immune inhibitors (*NFKBIA*) and chemokine receptors (*CCR4*) (fold change 1.3 and 1.4, respectively) (Supplementary Figure 4). When looking at the effects of infection on expression of immune-related genes in the proximal colon we found an upregulation of genes related to type-2 responses such as *CCL26*, *CCR4*, and *RETNLB* (fold changes 1.4, 1.5 and 1.4, respectively) (Supplementary Figure 5). Furthermore, there was a tendency for increased expression of *IL4* and *EPX* in infected pigs, regardless of diet (Table 5). However, infected pigs also had increased expression of the pro-inflammatory gene *STAT4* (fold change 1.2), and *IL12A, TLR5* and *CD86* expression tended to be increased. Most notably, we found that the ISL gene expression was consistently higher, relative to IC (Fig. 5), in contrast to the effect seen in the jejunum. Thus, we found significant interactions of diet and infection for several genes, especially those related to the mucosal barrier (Table 5), such as *DCLK1*, *HDAC9*, *IL13RA1*, *MUC2* and *HDAC1* (Fold change 2.5, 1.9, 1.3, 1.3 and − 1.2, respectively). The same effect was seen for pro-inflammatory genes *ILIB2*, *IL1R1*, *NFKB1*, *STAT3* (fold change 1.3, 1.7, 1.3 and 1.2, respectively)*,* and type-2 response genes *ARG1* and *CCL22* (Fold change 1.3 and 1.7, respectively) (Supplementary Figure 6).

**Table 5:** Relative expression and significance (P-value) of all genes influenced by diet, infection or their interaction in proximal colon tissue from groups of pigs (n = 8): control group (UC), *Oesphagostomum dentatum* and *Ascaris suum* infection (IC), fermented *Saccharina latissima* supplementation (USL), or *O. dentatum* and *A. suum* infection combined with fermented *S. latissima* supplementation (ISL). * indicates p ≤ 0.05, # indicates a trend of effect where p ≤ 0.1, ^ indicates close to the limit of detection, ^^ indicates variation within cDNA replicates (19% of the cDNA replicates varied more than 1.5 Cq).

Immune function	Immune gene	Relative expression				Significance (*P* value)		
		UC	IC	USL	ISL	Diet	Infection	Interaction
Th1	*IL1B2*	1.6	2.4	3.0	2.0			*0.04
	*IL1R1*	2.5	2.7	2.2	4.2			*0.01
	*IL6*	8.3	9.6	11.4	14.3	0.13		
	*IL7*	1.7	1.7	1.4	1.6	*0.05		
	*IL12A*	3.0	3.6	2.7	3.6		#0.06	
	*IL15*	3.3	3.7	2.8	3.6		0.11	
	*IL18*	2.1	2.1	1.6	2.5			#0.10
	*TLR1*	2.4	2.3	1.8	2.7			#0.06
	*TLR3*	2.4	2.4	1.8	2.3	0.10	0.13	
	*TLR5*	4.1	4.9	3.7	5.8		#0.09	
	*TLR7*	2.4	2.0	2.4	3.1			0.14
	*INOS*	7.3	4.6	7.4	9.7			#0.09
	*CD86*	1.5	1.7	1.5	1.9		#0.06	
	*STAT1*	2.9	2.7	2.6	3.4			0.14
	*STAT4*	1.7	2.0	1.6	2.0		*0.03	
	*PTGS2*	3.3	2.5	4.4	4.6	#0.06		
	*ICAM1*	3.2	2.7	3.2	4.2			0.11
*Th*	*CD4*	2.9	3.0	3.2	4.5	0.13		
Th2	*IL4*	1.7	2.2	1.9	2.4		#0.07	
	*ARG1*	56.7	139.6	388.2	75.5			*0.02
	*EPX^*	2.2	3.3	2.1	6.8		#0.07	
	*STAT6*	3.9	3.1	3.4	4.5			#0.07
	*CCL2*	1.6	2.0	1.9	2.3	0.11	#0.10	
	*CCL22*	2.2	1.8	2.4	3.6			*0.05
	*CCL26*	2.3	3.3	2.6	5.4	#0.09	*0.01	
	*CCR4*	4.0	5.9	5.6	10.4	*0.05	*0.01	
*Th17*	*STAT3*	1.7	1.3	1.5	2.1			*0.002
T-reg	*IL10*	1.7	2.2	2.4	2.6	0.12	0.10	
	*CCL19*	2.7	2.5	2.8	3.9			0.13
Mucusal barrier function	*DCLK1*	2.2	2.3	2.4	5.3			*0.004
	*HDAC1*	2.7	2.1	2.1	2.3			*0.05
	*HDAC6*	1.8	1.6	1.6	1.9			0.11
	*HDAC9*	2.0	1.8	1.6	3.7			*0.002
	*IL4R*	1.8	1.5	1.7	2.0			#0.08
	*IL13RA1*	2.0	2.0	1.7	2.6			*0.02
	*MUC2^^*	4.3	2.8	3.2	5.3			*0.02
	*RETNLB*	3.7	5.4	3.9	4.9		*0.04	
Innate	*NFKB1*	1.3	1.3	1.3	1.7			*0.04
	*NFKBIA*	1.3	1.5	1.7	1.7	*0.04		
	*TANK*	2.2	2.2	2.0	2.3			0.15
Lipid metabolism	*APOA1*	1.9	2.0	2.2	2.7	#0.10		
	*PPARG*	2.4	2.0	1.9	2.2			#0.09

Taken together, these data suggest that Fer-SL inclusion only had a slight regulatory effect on the immune genes in the intestinal tract in uninfected pigs, with decreased expression of some pro-inflammatory immune genes. However, during a helminth infection, the inclusion of Fer-SL induced an attenuation of immune genes in the jejunum and upregulation of immune genes in the proximal colon.

### Limited effects of seaweed or parasite infection on systemic immune parameters

Further investigations of the systemic immune-modulating effects of Fer-SL inclusion in diet were conducted on peripheral blood mononuclear cells (PBMCs) and cells from the ileo-caecal lymph nodes (CLN). In Study 2, we found slight modulatory effects of dietary Fer-SL inclusion on PBMCs, with a small reduction in the proportion of CD14+  onocytes within the PBMC population in the USL group compared to the UC group (− 7.7%, *P* = 0.07). Furthermore, we found a small increase in the proportion of CD3+ T-cells (8.8%, *P* = 0.05) (Supplementary Figure 7). Infection did not show any significant effects on PBMCs, apart from a reduced amount of CD14hi cells in infected pigs (*P* = 0.03) (Supplementary Figure 8). In the lymph nodes, we found no significant effects of Fer-SL diet inclusion, but an effect of infection, with a reduction in the proportion of CD3+ T-cells in infected vs non-infected animals (*P* = 0.03), and a small increase in CD14+ monocytes in infected vs non-infected animals, although this was not significant (*P* = 0.08). A significant difference was found between the UC and ISL group (*P* = 0.04) (Supplementary Figure 9). Ex vivo stimulation of PBMCs showed no significant differences in LPS-induced TNF-α or IL-1β secretion in the different treatment groups (data not shown).

Overall, Fer-SL inclusion showed very limited effects on the systemic immune response to helminths with only a trend of a decrease of CD14+ monocytes and an increase of CD3+ T-cells in PBMCs.

## Discussion

Here, we investigated the effects of seaweed dietary inclusion during helminth infection in pigs. It is normally advised to include seaweed at low levels (1–2%) in pig feed to achieve the beneficial prebiotic effects without adverse effects, such as weight loss^2,53^, and avoid high levels of trace elements, such as iodine^4^. However, our study highlights that there were no apparent adverse effects on growth or health at short-term inclusion levels of 5 or 8%. Although *S. latissima* showed promising effects on egg excretion at a 5% inclusion level of both Fer-SL and Non-Fer-SL, it did not significantly affect worm burdens and egg excretion at 8% dietary inclusion of Fer-SL. The reason for the lack of effect on worm burden in vivo with Fer-SL inclusion, despite promising results in vitro, could be that the omega-3 fatty acids, which were the active compounds found in vitro^28^, were not able to reach the nematodes in high enough concentrations locally. This could be explained by the metabolism of fatty acids, as free fatty acids are quickly absorbed and metabolised^54^. Another factor could be interactions with the digesta, which have previously been shown to reduce the effects of natural dietary compounds against *A. suum* migration^55^. The reasons for an apparent discrepancy between the preliminary data and the larger trial may be numerous. Potentially, minor differences in the fermentation efficacy between the two diets, small differences in feed intake, or differences related to pig cohorts, e.g. basal GM composition in the two studies may have contributed.

Despite not showing significant anti-parasitic effects, Fer-SL inclusion had a modulatory effect on GM composition with an increase in both Observed features and Shannon index diversity in the GM of the proximal colon. Our data have to be interpreted with some caution as pigs were group-housed, which allowed normal social behaviour but does induce a confounding factor of pen effect. However, we found consistent GM changes in the two pens fed Fer-SL (regardless of infection status), and the results of GM changes are in accordance with previous studies on the effect of seaweed-derived components on the pig GM, which have also found decreases in *Enterobacteriaceae* (Proteobacteria) ^7^. Collectively, the GM analysis showed that there was limited effect of parasite infection on the GM composition, regardless of dietary treatment, and that Fer-SL inclusion was the driving factor behind the changes in altered GM diversity. These changes were manifested by the increase in the abundance of taxa which have been putatively linked to nutrient digestibility (e.g., *Anaeroplasma* and *Treponema*)^56^, butyrate production by fermentation of seaweed components (e.g., *Oscillospira*^57^ and *Clostridiales*^58^), and the seaweed epiphytic bacterial communities (*Desulfovibrionaceae*)^59^. Furthermore, the abundance of the bacteria *Prevotella copri* was reduced. Some studies have found this bacteria associated with modified mucus layer permeability^60^ and increased sensitivity to chemically induced colitis in mice^61^. However, a positive correlation between *Prevotella* spp. on production and health in pigs has also been found^62^. Taken together, these data show that Fer-SL was able to modulate GM composition and increase the relative abundance of taxa putatively associated with gut health. Importantly, these changes were also apparent in helminth-infected pigs, indicating a stable and robust prebiotic effect of Fer-SL in pigs either with or without an acute enteric infection.

Pig feeding trials with compounds from brown seaweeds have in general been found to stimulate *Lactobacillus* growth and reduce *Enterobacteriaceae* or *E.coli*^4^, and laminarin from *L. digitata* has been found to decrease *Enterobacteriaceae* in the colon^63,64^. The GM modulating effect of including Fer-SL in the diet could have been induced by polysaccharides, such as laminarin, working as dietary fibres^65^, but inhibitory effects of *S. latissima* extract directly on bacteria have also been found^66^, indicating a direct effect of *S. latissima* on the gut bacteria. Interestingly, we did not see any significant effects on GM composition by the parasite infections, even though many helminth infections may result in expansion of e.g. lactobacilli^12^*.* A recent study found infection with *A. suum* reduced *Lactobacillus* and *Ruminicoccus*, and gave non-significant increases in *Prevotella*^55^, unlike what was seen in our study.

We speculate that the observed lack of effect on the GM by infection could be due to the co-infection with two parasites (*A. suum* and *O. dentatum*). Previous studies have found that *O. dentatum* is able to change the immune response toward other parasites during co-infection^67,68^. Therefore, it can be speculated that an interaction between the two parasites on GM is occurring, and the co-infection of the two parasites may cancel out each other's effects on the GM. Although, one study indicates that *O. dentatum* may be able to suppress the effects of probiotics on GM^69^, studies of the effects of *O. dentatum* infection on the GM are scarce.

Brown seaweeds, such as *S. latissima*, contain storage polysaccharides such as laminarin^1,70^, and seaweed polysaccharides can be fermented by the GM in the large intestine^71^. However, the *S. latissima* we used was fermented pre-feeding, thus it may be likely that the bioactivity we observed on gene expression and GM composition derives from fermentation products present in the diet, rather than active metabolism of the seaweed polysaccharides in the gut. It is currently unclear which active compounds within seaweed lead to the modulatory effects on the GM and immune function, and whether they are produced in the pre-feeding fermentation or in vivo following seaweed metabolism. Identification of such compounds could lead to a more standardized approach to design seaweed-based supplements that have specific health benefits. However, it should be noted that the biological activity is unlikely to derive from a single compound, and instead is most probably due to a synergistic interaction between diverse bioactive molecules, which may present some difficulties for standardization.

In general, Fer-SL inclusion tended to decrease expression of pro-inflammatory genes in both the jejunum and proximal colon and increase expression of type-2 response genes in the proximal colon. Seaweed extract inclusion in the feed has been found to downregulate Th1 pro-inflammatory genes such as *IL1B* and *IL6*, but also *IL17A*^7^, and ex vivo results with ethanol extracts on porcine colonic tissue also showed decrease in *IL6*, *IL10,* and *CCL2* expression*,* as well as an increase in *APO1* expression^72^. Cytokine gene expression of pro-inflammatory genes *IL1α*, *IL10*, *TNFα* and *IL17A* was down-regulated in the colon following exposure to laminarin from *L. digitata, L. hyperborea and Saccharomyces cerevisiae*, and a significant increase in *IL8* gene expression in the colon following an LPS challenge ex vivo when exposed to laminarin from *L. digitata*^63^. However, these studies were carried out using different species of brown seaweed and pigs of a different age group compared to the current study. Infection also affected the gene expression in the intestinal segments, with an increase in expression of type-2 associated genes in the colon and jejunum, which is in line with previous studies showing increases in type-2 response-related genes with both *Ascaris* and *Oesophagostomum* infections in pigs^33,67^. Fer-SL appeared to modulate gene expression during infection, albeit with relatively modest fold changes. Thus, it is not clear whether Fer-SL or related dietary interventions can modulate immune function and/or reduce inflammation during mucosal pathogen infection. However, such immune-modulation would have important implications for animal production in an era of reduced antimicrobial drug usage, and thus further studies to assess whether Fer-SL administered under large scale, commercial conditions may impact immune function and disease resistance are warranted.

Fer-SL inclusion by itself tended to positively affect gut morphology, with increased villi/crypt ratios in the jejunum in uninfected pigs (UC vs USL), unlike what was found in a study with piglets offered *L. digitata* extract, which resulted in reduced villus height in the small intestine^9^. Reduced villi height has been found to have negative effects on absorption capability^73^. We also saw that goblet cells in the jejunum tended to increase with Fer-SL inclusion in non-infected pigs (UC vs USL), which correlates well with a higher abundance of the mucus encoding gene *MUC2* in pigs feed seaweed extracts, despite it originating from another seaweed, *Laminaria* spp.^10,11,64^.

## Conclusion

Inclusion of 8% Fer-SL in the diet of growing pigs did not significantly alter parasite burdens or associated pathology. However, Fer-SL inclusion showed a moderate local modulatory effect on the immune system, with general down-regulation of pro-inflammatory genes and upregulation of type 2 response genes. Interestingly, Fer-SL induced an overall decrease in the expression of immune-related genes in the jejunum of infected pigs. In contrast, the expression of immune regulating genes was increased in colon tissue during infection. In addition, microbial composition in the proximal colon was not distinctly affected by infection, but Fer-SL inclusion did in both groups show significantly increased diversity of the GM in the proximal colon, increasing the relative abundance of taxa putatively associated with gut health. These results indicate Fer-SL inclusion, despite not displaying any substantial anti-parasitic effects, may modulate GM composition and hence potentially also gut health regardless of the presence of intestinal parasites.

## Ethical approval

All experimentation was approved by the Danish Animal Experimentation Inspectorate (License number 2015-15-0201-00760), and conducted at the Experimental Animal Unit, University of Copenhagen according to FELASA guidelines and recommendations. The study is reported in accordance with ARRIVE guidelines (https://arriveguidelines.org).

### Supplementary Information

Supplementary Information.

## Data Availability

16S rRNA gene amplicon sequencing raw data are available at Sequence Read Archive (www.ncbi.nlm.nih.gov/sra/) under the accession number PRJNA817076.

## References

[1] Holdt SL, Kraan S (2011). Bioactive compounds in seaweed: functional food applications and legislation. J. Appl. Phycol..

[2] Makkar HPS (2016). Seaweeds for livestock diets: A review. Anim. Feed Sci. Technol..

[3] Øverland M, Mydland LT, Skrede A (2019). Marine macroalgae as sources of protein and bioactive compounds in feed for monogastric animals. J. Sci. Food Agric..

[4] Corino C, Modina SC, Di Giancamillo A, Chiapparini S, Rossi R (2019). Seaweeds in pig nutrition. Animals.

[5] Dierick N, Ovyn A, De Smet S (2009). Effect of feeding intact brown seaweed *Ascophyllum nodosum* on some digestive parameters and on iodine content in edible tissues in pigs. J. Sci. Food Agric..

[6] Shimazu T (2019). Addition of Wakame seaweed (*Undaria pinnatifida*) stalk to animal feed enhances immune response and improves intestinal microflora in pigs. Anim. Sci. J..

[7] Walsh AM, Sweeney T, O'Shea CJ, Doyle DN, O'Doherty JV (2013). Effect of dietary laminarin and fucoidan on selected microbiota, intestinal morphology and immune status of the newly weaned pig. Br. J. Nutr..

[8] Lynch MB, Sweeney T, Callan JJ, O'Sullivan JT, O'Doherty JV (2010). The effect of dietary *Laminaria* derived laminarin and fucoidan on intestinal microflora and volatile fatty acid concentration in pigs. Livest. Sci..

[9] Reilly P (2008). The effects of seaweed extract inclusion on gut morphology, selected intestinal microbiota, nutrient digestibility, volatile fatty acid concentrations and the immune status of the weaned pig. Animal.

[10] Leonard SG, Sweeney T, Bahar B, Lynch BP, O'Doherty JV (2011). Effects of dietary seaweed extract supplementation in sows and post-weaned pigs on performance, intestinal morphology, intestinal microflora and immune status. Br. J. Nutr..

[11] Ryan MT (2010). Effects of nutrient supplementation with laminarin derived from *Laminaria hyperborea* and *Laminaria digitata* on mucin gene expression in the porcine ileum. Livest. Sci..

[12] Leung JM, Graham AL, Knowles SCL (2018). Parasite-Microbiota interactions with the vertebrate gut: Synthesis through an ecological lens. Front. Cell. Infect. Microbiol..

[13] Williams AR (2021). Emerging interactions between diet, gastrointestinal helminth infection, and the gut microbiota in livestock. BMC Vet. Res..

[14] Kaplan RM, Vidyashankar AN (2012). An inconvenient truth: global worming and anthelmintic resistance. Vet. Parasitol..

[15] Roepstorff A, Bjørn H, Nansen P (1987). Resistance of *Oesophagostomum* spp. in pigs to pyrantel citrate. Vet. Parasitol..

[16] Gerwert S, Failing K, Bauer C (2002). Prevalence of levamisole and benzimidazole resistance in *Oesophagostomum* populations of pig-breeding farms in North Rhine-Westphalia, Germany. Parasitol. Res..

[17] Macrelli M (2019). First detection of ivermectin resistance in *Oesophagostomum dentatum* in pigs. Vet. Parasitol..

[18] Dangolla A, Bjørn H, Willeberg P, Barnes EH (1997). Faecal egg count reduction percentage calculations to detect anthelmintic resistance in *Oesophagostomum* spp. in pigs. Vet. Parasitol..

[19] Pettersson E (2021). First report on reduced efficacy of ivermectin on *Oesophagostomum* spp. on Swedish pig farms. Vet. Parasitol. Reg. Stud. Rep..

[20] Petkevicius S (1995). The effect of two types of diet on populations of *Ascaris suum* and *Oesophagostomum dentatum* in experimentally infected pigs. Parasitology.

[21] Jensen AN (2011). The effect of a diet with fructan-rich chicory roots on intestinal helminths and microbiota with special focus on *Bifidobacteria* and *Campylobacter* in piglets around weaning. Animal.

[22] Hoste H (2015). Tannin containing legumes as a model for nutraceuticals against digestive parasites in livestock. Vet. Parasitol..

[23] Peña-Espinoza M, Thamsborg SM, Desrues O, Hansen TV, Enemark HL (2016). Anthelmintic effects of forage chicory (*Cichorium intybus*) against gastrointestinal nematode parasites in experimentally infected cattle. Parasitology.

[24] Hussain A (2016). Fermentation, a feasible strategy for enhancing bioactivity of herbal medicines. Food Res. Int..

[25] Puupponen-Pimiä R (2016). Fermentation and dry fractionation increase bioactivity of cloudberry (*Rubus chamaemorus*). Food Chem..

[26] Jørgensen H, Sholly D, Pedersen AØ, Canibe N, Knudsen KEB (2010). Fermentation of cereals—Influence on digestibility of nutrients in growing pigs. Livest. Sci..

[27] Lyberg K, Lundh T, Pedersen C, Lindberg JE (2006). Influence of soaking, fermentation and phytase supplementation on nutrient digestibility in pigs offered a grower diet based on wheat and barley. Anim. Sci..

[28] Bonde CS (2021). Bio-guided fractionation and molecular networking reveal fatty acids to be principal Anti-Parasitic compounds in Nordic seaweeds. Front. Pharmacol..

[29] Vils, E. Håndbog i svinehold (Landsbrugforlaget 2006).

[30] Roepstorff A, Nansen P (1998). Epidemiology, diagnosis and control of helminth parasites of swine. FAO Anim. Health Man..

[31] Roepstorff A, Eriksen L, Slotved HC, Nansen P (1997). Experimental *Ascaris suum* infection in the pig: Worm population kinetics following single inoculations with three doses of infective eggs. Parasitology.

[32] Andersen-Civil AIS (2022). Dietary proanthocyanidins promote localized antioxidant responses in porcine pulmonary and gastrointestinal tissues during *Ascaris suum*-induced type 2 inflammation. FASEB J..

[33] Dawson HD (2005). Localized multigene expression patterns support an evolving Th1/Th2-like paradigm in response to infections with *Toxoplasma gondii* and *Ascaris suum*. Infect. Immun..

[34] Masure D (2013). the intestinal expulsion of the roundworm *Ascaris suum* is associated with eosinophils, intra-epithelial T cells and decreased intestinal transit time. PLoS Negl. Trop. Dis..

[35] Slotved HC (1997). Use of an agar-gel technique for large scale application to recover *Ascaris suum* larvae from intestinal contents of pigs. Acta Vet. Scand..

[36] Slotved HC (1996). Recovery of *Oesophagostomum dentatum* from pigs by isolation of parasites migrating from large intestinal contents embedded in agar-gel. Vet. Parasitol..

[37] Myhill LJ (2018). Mucosal barrier and Th2 immune responses are enhanced by dietary inulin in pigs infected with *Trichuris suis*. Front. Immunol..

[38] Skovgaard K (2009). Rapid and widely disseminated acute phase protein response after experimental bacterial infection of pigs. Vet. Res..

[39] De Coster W, D'Hert S, Schultz DT, Cruts M, Van Broeckhoven C (2018). NanoPack: Visualizing and processing long-read sequencing data. Bioinformatics (Oxford, England).

[40] Caporaso JG (2010). QIIME allows analysis of high-throughput community sequencing data. Nat. Methods.

[41] McDonald D (2012). An improved Greengenes taxonomy with explicit ranks for ecological and evolutionary analyses of bacteria and archaea. ISME J.

[42] Bolyen E (2019). Reproducible, interactive, scalable and extensible microbiome data science using QIIME 2. Nat. Biotechnol..

[43] R: A language and environment for statistical computing [Internet]. (R Foundation for Statistical Computing, Vienna, Austria, 2020. https://www.r-project.org/.

[44] R: a language and environment for statistical computing (R Foundation for Statistical Computing, 2020. https://www.r-project.org/.

[45] McMurdie PJ, Holmes S (2013). phyloseq: an R package for reproducible interactive analysis and graphics of microbiome census data. PLoS ONE.

[46] Wickham H (2019). Welcome to the Tidyverse. J. Open Source Softw..

[47] “ggplot2” Based Publication Ready Plots. 2020. https://cran.r-project.org/package=ggpubr.

[48] Wickham H (2007). Reshaping data with the reshape package. J. Stat. Softw..

[49] Love MI, Huber W, Anders S (2014). Moderated estimation of fold change and dispersion for RNA-seq data with DESeq2. Genome Biol..

[50] Pretty Heatmaps. 2019. https://cran.r-project.org/package=pheatmap.

[51] ColorBrewer Palettes. 2014. https://cran.r-project.org/package=RColorBrewer.

[52] RVAideMemoire: Testing and Plotting Procedures for Biostatistics. 2021. https://cran.r-project.org/package=RVAideMemoire.

[53] Evans FD, Critchley AT (2014). Seaweeds for animal production use. J. Appl. Phycol..

[54] Christensen MS, Høy CE, Becker CC, Redgrave TG (1995). Intestinal absorption and lymphatic transport of eicosapentaenoic (EPA), docosahexaenoic (DHA), and decanoic acids: Dependence on intramolecular triacylglycerol structure. Am. J. Clin. Nutr..

[55] Williams AR (2017). A polyphenol-enriched diet and *Ascaris suum* infection modulate mucosal immune responses and gut microbiota composition in pigs. PLoS ONE.

[56] Niu Q (2015). Dynamic distribution of the gut microbiota and the relationship with apparent crude fiber digestibility and growth stages in pigs. Sci. Rep..

[57] Gophna U, Konikoff T, Nielsen HB (2017). Oscillospira and related bacteria: From metagenomic species to metabolic features. Environ. Microbiol..

[58] Levine UY, Looft T, Allen HK, Stanton TB (2013). Butyrate-producing bacteria, including mucin degraders, from the swine intestinal tract. Appl. Environ. Microbiol..

[59] Selvarajan R (2019). Distribution, interaction and functional profiles of epiphytic bacterial communities from the rocky intertidal seaweeds, South Africa. Sci. Rep..

[60] Rolhion N (2019). A *Listeria monocytogenes* bacteriocin can target the commensal *Prevotella copri* and modulate intestinal infection. Cell Host Microbe.

[61] Scher JU (2013). Expansion of intestinal *Prevotella copri* correlates with enhanced susceptibility to arthritis. eLife.

[62] Amat S, Lantz H, Munyaka PM, Willing BP (2020). *Prevotella* in pigs: The positive and negative associations with production and health. Microorganisms.

[63] Sweeney T (2012). Effect of purified β-glucans derived from *Laminaria digitata*, *Laminaria hyperborea* and *Saccharomyces cerevisiae* on piglet performance, selected bacterial populations, volatile fatty acids and pro-inflammatory cytokines in the gastrointestinal tract of pigs. Br. J. Nutr..

[64] Smith AG (2011). The effects of laminarin derived from *Laminaria digitata* on measurements of gut health: Selected bacterial populations, intestinal fermentation, mucin gene expression and cytokine gene expression in the pig. Br. J. Nutr..

[65] Gupta S, Abu-Ghannam N (2011). Bioactive potential and possible health effects of edible brown seaweeds. Trends Food Sci. Technol..

[66] Cox S, Abu-Ghannam N, Gupta S (2010). An assessment of the antioxidant and antimicrobial activity of six species of edible Irish seaweeds. Int. Food Res. J..

[67] Andreasen A (2015). Immune and inflammatory responses in pigs infected with *Trichuris suis* and *Oesophagostomum dentatum*. Vet. Parasitol..

[68] Petersen HH, Andreasen A, Kringel H, Roepstorff A, Thamsborg SM (2014). Parasite population dynamics in pigs infected with *Trichuris suis* and *Oesophagostomum dentatum*. Vet. Parasitol..

[69] Myhill LJ (2022). Parasite-probiotic interactions in the gut: *Bacillus* sp. and *Enterococcus faecium* regulate type-2 inflammatory responses and modify the gut microbiota of pigs during helminth infection. Front. Immunol..

[70] Schiener P, Black KD, Stanley MS, Green DH (2015). The seasonal variation in the chemical composition of the kelp species *Laminaria digitata*, *Laminaria hyperborea*, *Saccharina latissima* and *Alaria esculenta*. J. Appl. Phycol..

[71] You L (2020). Beneficial effects of three brown seaweed polysaccharides on gut microbiota and their structural characteristics: An overview. Int. J. Food Sci. Technol..

[72] Bahar B, O'Doherty JV, Smyth TJ, Sweeney T (2016). A comparison of the effects of an *Ascophyllum nodosum* ethanol extract and its molecular weight fractions on the inflammatory immune gene expression *in-vitro* and *ex-vivo*. Innov. Food Sci. Emerg. Technol..

[73] Nabuurs MJ, Hoogendoorn A, van der Molen EJ, van Osta AL (1993). Villus height and crypt depth in weaned and unweaned pigs, reared under various circumstances in The Netherlands. Res. Vet. Sci..

