# Supplementary material for: Dietary seaweed (Saccharina latissima) supplementation in pigs induces localized immunomodulatory effects and minor gut microbiota changes during intestinal helminth infection
Source: Sci Rep. 2023 Dec 11;13:21931. doi: 10.1038/s41598-023-49082-5 (PMC10713666; doi:10.1038/s41598-023-49082-5)
Supplement: Supplementary file 1 — Supplementary Information. [file 41598_2023_49082_MOESM1_ESM.pdf]

Supplementary Material

Dietary seaweed (*Saccharina latissima*) supplementation modifies the gut microbiota and induces localized immunomodulatory effects during intestinal helminth infection in pigs

Charlotte Smith Bonde[1], Helena Mejer[1], Laura J. Myhill[1], Ling Zhu[1], Penille Jensen[1], Nilay Büdeyri Gökgöz[2], Lukasz Krych[2], Dennis Sandris Nielsen[2], Kerstin Skovgaard[3], Stig Milan Thamsborg[1], Andrew R. Williams[1*]

[1]*Department of Veterinary and Animal Sciences, University of Copenhagen, Frederiksberg, Denmark,*

[2]*Department of Food Science, University of Copenhagen, Frederiksberg, Denmark,*

[3]*Department of Biotechnology and Biomedicine, Section for Protein Science and Biotherapeutics, Technical University of Denmark, Kongens Lyngby, Denmark.*

**Corresponding author - arw@sund.ku.dk*

Supplementary Table 1: Primer sequences used for Fluidigm gene expression analysis

Gene of interest	Sequence (5' to 3')	Sequence (5' to 3')	Amplicon length
ACTB (Actin, Beta)	F: GGATGCAGAAGGAGATCACG	R: ACACGGAGTACTTGCGCTCT	84
APOA1 (Apolipoprotein A-I)	F:GTTCTGGGACAACCTGGAAA	R: GCTGCACCTTCTTCTTCACC	86
ARG1 (Arginase)	AATTGGCAAGGTGATGGAAG	TCCAGTCCATCCACATCAAA	90
B2M (Beta-2-Microglobulin	F: TGAAGCACGTGACTCTCGAT	R: CTCTGTGATGCCGGTTAGTG	70
CCL17 (C-C Motif Chemokine Ligand 17))	GGGTGGTACCAGACCTCAGA	GTCCTTGGGGTCAGAACAGA	90
CCL19	F: CTGGACTTCTCCTGCTCTGG	R: AAAGGCTCGAACCAGATTCC	97
CCL2	F:CTTCTGCACCCAGGTCCTT	R: CGCTGCATCGAGATCTTCTT	93
CCL22	F: CCCTGCGTGTGGTGAAGTAT	R: ATCTCTCGGTCCCTCAAGGT	88
CCL26	F: CTGCTTCCAATACAGCCACA	R: AGCAGCTGTTCCTGGTGAAT	74
CCR1 (C-C Motif Chemokine Receptor 1)	CTGGCCATTTCTGACCTGAT	ACACATGTGATCGCCAAAAA	93
CCR3	CCAGCTGTGAACAGAGCAAA	GGCATAGATTACGGGGTTGA	95
CCR4	ACACCTGGACTACGCCATTC	TTTCTCCCCCAGGAAGAAGT	91
CCR5	GCCGCAATGAGAAGAAGAAG	AGGGAGCCCAGAAGAGAAAG	81
CCR7	TCCACGTCTGCAAACTCATC	GTCGATGCTGATGCAGAGAA	83
CD163	GGAAGTGAGCAGGTCTGGAG	ACTCTGGTTCCCTGAGCAGA	130
CD4	GGTCGAGGTTCTGCCCA	AGAACCCAGCGAGAAACAGA	98
CD40	F: TGAGAGCCCTGGTGGTTATC	R: GCTCCTTGGTCACCTTTCTG	90
CD86	F: CATCGTCTGTGTCCTGCAAC	R: CACAGGTGGCTTTGCATCTA	82
CXCL10 (Chemokine (C-X-C Motif) Ligand 10)	F:CCCACATGTTGAGATCATTGC	R: GCTTCTCTCTGTGTTCGAGGA	141
CXCL9	F: AGCAGTGTTGCCTTGCTTTT	R: ATGCAGGAACAACGTCCATT	92
CXCR4 (Chemokine (C-X-C Motif) Receptor 4)	ACGGGTTCCGTATATTCACTTC	GGAAACAGGGTTCCTTTATGG	87
DCLK1 (Doublecortin Like Kinase 1)	GACCGATTCCGATCTTTTGA	CTGGGGCAAATTCACGTTAT	75
EPX (Eosinophil peroxidase)	TTGGCAACTGAACTGAGACG	CAGAAAGTCCCGGTAGGTGA	83
FFAR (free fatty acid receptor 1 or GPR40)	ACCCTACAATGCCTCCAATG	CTCCAAGCACCTGTGATGAG	90
FFAR2	TGCCTCGTCCTCTTCTTCAT	CGAGCATGATCCACACAAAG	73
FOXP3 (Forkhead Box P3)	F: GAAGGACAGCACCCTTTCAA	R: AGGAAGTCCTCTGGCTCCTC	111
GAPDH (Glyceraldehyde-3-Phosphate Dehydrogenase)	CCCTCAAGATCGTCAGCAAT	GTCATGAGTCCCTCCACGAT	87
GATA3 (GATA Binding Protein 3)	F: ACCCCTTATTAAGCCCAAGC	R: TCCAGAGAGTCGTCGTTGTG	92
GZMA (Granzyme A)	GAAGCCTGGCGTCTACATTC	TATGGCTCCTGCAATGGTTT	73
GZMB	F: CCAGGACCAGGATAATCGAA	R: GGGTGACGTTGATTGAGCTT	101
ICAM1 (Intercellular Adhesion Molecule 1)	F:AAGCTTCTCCTGCTCTGCTG	R: GGGGTCCATACAGGACACTG	89
ICAM2	CGGACACCTCATTCACAGAG	TGCCACAAACAAGAAGAGCA	121
IFNG (Interferon gamma)	F:CCATTCAAAGGAGCATGGAT	R: TTCAGTTTCCCAGAGCTACCA	76
IL10 (Interleukin 10)	CTGCCTCCCACTTTCTCTTG	TCAAAGGGGCTCCCTAGTTT	95
IL12A (Interleukin 12 p35)	GACAACCCTGTGCCTTAGC	CAGAAGCTTTGCATTCATGG	82
IL12B (Interleukin 12 p40)	F: GACCAGAAAGAGCCCAAAAAC	R: AGGTGAAACGTCCGGAGTAA	70
IL13	GAAGACACCCCTATGCAACG	GCAGTCGGAGATGTTGATGA	100
IL13	F: CCAAGCGAGCAAGTTCCTG	R: AACTACCCGTGGCGAAAAAT	110
IL13RA1 (Interleukin 13 Receptor Subunit Alpha 1)	CAATGCGGGAAAAATCAGAC	TATGCGGAGGATCAGGTTTC	74
IL15	GATGCTCATCCCAATTGCAA	TGACGCGTAACTCCAGGAGAA	71
IL18	F:CTGCTGAACCGGAAGACAAT	R: TCCGATTCCAGGTCTTCATC	100
IL1A (Interleukin 1 Alpha)	F: TGTGCTAAATAACCTGGATGAGG	R: GGTTCGTCTTCGTTTTGAGC	135
IL1B (Interleukin 1, Beta)	F:TCTCTCACCCCTTCTCCTCA	R: GACCCTAGTGTGCCATGGTT	60
IL1R1 (Interleukin 1 Receptor Type 1)	F: CTCCCGGGTGATAAAACTGA	R: CCCAAGATCACTTCCATCGT	96
IL23A (interleukin 23)	F: GCTGTGATCCTCAGGGACTC	R: TAGAGAAGGCTCCCCTGTGA	119
IL33	F:GTAAACCTGAGCCCCACAAA	R: CTGTTCTGGCAGTGGGTTTT	102
IL4	ACTGATCCCAACCCTGGTCT	GTGATGTCGCACTTGTGTCC	72
IL4	F: GCAAACATGACCTGTTCTGTG	R: GCTTCAACACTTTGAGTATTTCTCC	105
IL4R (Interleukin 4 Receptor)	CTGGCACTCTTGGGTGTCTC	TGGAGCTCTGAACATTGCTG	79
IL5	F: TGCCTACGTTAGTGCCATTG	R: TCGATGAATGGAGAGCAGTG	82

IL6	F:CCTCTCCGGACAAAACTGAA	R: TCTGCCAGTACCTCCTTGCT	118
IL7	CCAACTCACTAAGAACTTGGAAGA	TCAATATTCTTTAGCACCCCTCA	139
IL8	TTGCCAGAGAAATCACAGGA	TGCATGGGACACTGGAAATA	78
MUC1 (Mucin 1, Cell Surface Associated)	GGCAGTACCAAACGGAACC	CAGGGCGATGACATAGATGA	137
MUC2	F: GCACGTCTGCAACAAGGAC	R: CAAAGCCCTCCAGGCAGT	125
MUC5AC (Mucin 5AC, Oligomeric Mucus/Gel-Forming)	F: CCCAGATCTGCAGCACCTAC	R: GTAACACAGGCCACCTGCTT	94
NFKB1 (Nuclear Factor Kappa B Subunit 1)	TCCACAAGGCAGCAAATAGA	AAGCTGAGTTTGCGAAAGGA	83
NFKBIA (NFKB Inhibitor Alpha)	TGTCTTTGGGTGCTGATGTC	AGCCCCACACTTCAACAAGA	116
NFKBIA (Nuclear Factor Of Kappa Light Polypeptide Gene Enhancer In B-Cells Inhibitor, Alpha)	F:GAGGATGAGCTGCCCTATGAC	R: CCATGGTCTTTTAGACACTTTCC	85
PPARG (peroxisome proliferator-activated receptor gamma 2)	TGCTGTGGGGATGTCTCATA	CTGCCAACAGCTTCTCCTTC	74
RETNLB (Resistin Like Beta)	TAATCGCAAGGGGTTCTCAG	CTTGGAGCAGAGGGATTGAG	97
RPL13A (Ribosomal protein L13a)	F: ATTGTGGCCAAGCAGGTACT	R: AATTGCCAGAAATGTTGATGC	76
SCD (Stearoyl-CoA Desaturase)	GGCATTCCAGAATGACGTTT	GTGGGGATCAGCATCTGTTT	82
SLC1A2 (Solute Carrier Family 1 Member 2)	TAAGCCTCACAGCCACACTG	TGTCAGAATGAGGAGCATGG	83
SLC2A1	AGAAATATTGCGGCACCAAG	AATCCATTGGGCATGAGTTC	88
SLC2A2(Solute Carrier Family 2 (Facilitated Glucose Transporter), Member 2)	F: CATGTCAGTGGGACTTGTGC	R: TGGCCCAATTTCAAAGAAAC	100
STAT1(Signal Transducer And Activator Of Transcription 1)	F: CCTTGCAGAATAGAGAACATGATAC	R: CCTTTCTCTTGTTGTCAAGCATT	108
STAT1	CACGAAGGTGATGAACATGG	TCTTTCAGTTGCAGGTGTCG	75
STAT3	GAAGCTGACCCAGGTAGTGC	GGCAGGTCAATGGTATTGCT	89
STAT4	F: GAAAGCCACCTTGGAGGAAT	R: ACAACCGGCCTTTGTTGTAG	100
STAT6	GGCAGAAGAGAGTTGCCTGA	CAAGGGTGGGAACATGTCTT	82
TANK (TRAF family member-associated NFKB activator)	TCCAGAAAGACCCACTGTCC	AAGTTTCCCGGGTTGTCTCT	93
TBP (TATA box binding protein)	F: ACGTTCGGTTTAGGTTGCAG	R: CAGGAACGCTCTGGAGTTCT	96
TFF2 (Trefoil Factor 2)	F: GCTGCTTCGACTCCCAAGT	R: CATGACGCACTCCTCAGACT	80
TFF3	F: TGTTCTGGCTGCTAGTGGTG	R: CAGTCCACCCTGTCCTTGG	112
TLR1 (Toll Like Receptor 1)	GAATTTCTGGGGTTGAGTGC	GGGTCTTCTCTTTCCCCGTA	119
TLR2	F: CGGAGGTTGCATATTCCACAG	R: TGTGAAAGGGAACAGGGAAC	128
TLR3	ACATCTACTGAAAGATCCATTGTGC	TCTTCGCAAACAGAGTGCAT	148
TLR4	CAATAGCTTCTCCAGCTTTCCAG	TGGATAGGATTTCCCGTCAG	132
TLR5	AGTTCTGAACCTGGCCTTCA	TAAGCGAGCTTAGGCAGTCC	144
TLR7	GGAAATAGCATCAGCCAAGCTC	TTCCAGGTTGCGTAGCTCTT	132
TLR8	F: GCAAAGACCACCACCAACTT	R: ATCCGTCAGTCTGGGAATTG	129
TNF (Tumor Necrosis Factor)	F:CACGTTGTAGCCAATGTCAAAG	R: GAGGTACAGCCCATCTGTCG	129
CTLA4 (Cytotoxic T-lymphocyte protein 4 precursor)	CTCCTGTACCCACCACCCTA	AGAATCTGGGCATGGTTCTG	84
HDAC1 (histone deacetylase 1)	GGATCGGTTAGGTTGCTTCA	CCTCCCAGCATCAACATAGG	96
HDAC6	CCCAAATCCATCGCAGATAC	GGCGAACGACTTAGAACTGG	86
HDAC9	GAACAGATGCGACAGCAAAA	CTTTTGTTGCCAAGGGAGAC	76
TSLP (thymic stromal lymphopoietin)	CTGTGATGATAGGCCGGATT	TTTGGCAAATTCTTCCTTGG	88
INOS (NOS2)	CAGCCCAAGGTCTATGTTCAAG	ATAGAGGTGGCCTTGCTCCT	90
TLR9	CCTTTGGAGAAGACCCCACT	ACCTGCACCAGGAGAGAAAG	92
PTGS2 (prostaglandin-endoperoxide synthase 2)	GAACTTACAGGAGAGAAGGAAATGG	TTTCTACCAGAAGGGCAGGA	94

Supplementary Table 2: Primer sequences used for 16S amplicon analysis

Primers	Primer Sequence
UMI_338Fa	5’- GTCTCGTGGGCTCGG- NNNNNNNNNNNNNNN - ACWCCTACGGGWGGCAGCAG-3’
UMI_338Fb	5’- GTCTCGTGGGCTCGG- NNNNNNNNNNNNNNN - GACTCCTACGGGAGGCWGCAG-3’
UMI_27Fa	5’- GTCTCGTGGGCTCGG- NNNNNNNNNNNNNNN - AGAGTTTGATYMTGGCTYAG-3’
UMI_27Fb	5’- GTCTCGTGGGCTCGG- NNNNNNNNNNNNNNN - AGGGTTCGATTCTGGCTCAG-3’
UMI_1540R	5’- GTCTCGTGGGCTCGG- NNNNNNNNNNNNNNN - TACGGYTACCTTGTTACGACT-3’
UMI_1391R	5’- GTCTCGTGGGCTCGG- NNNNNNNNNNNNNNN - GACGGGCGGTGTGTRCA-3’

A

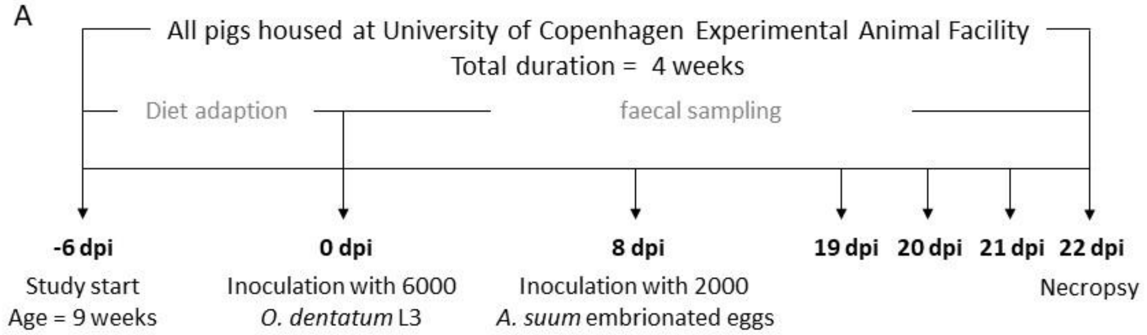

B

	**Control diet**	**Non-Fer-SL diet** (5% non-fermented *S. latissima*)	**Fer-SL diet** (5% fermented *S. latissima*)
***O. dentatum* and *A. suum* infection**	C<br>n = 3	NSL<br>n = 3	FSL<br>n = 3

Supplementary Figure 1: Timeline and study design of Study 1. (A) At arrival (-6 dpi, age 9 weeks) nine pigs were stratified according to weight and sex, and randomly allocated into three treatments groups (n=3). (B) All pigs were all infected with *Oesphagostomum dentatum* and *Ascaris suum* and fed a control diet or a diet with 5% (w/w) non-fermented (Non-Fer-SL) or fermented *Saccharina latissma* (Fer-SL).

A

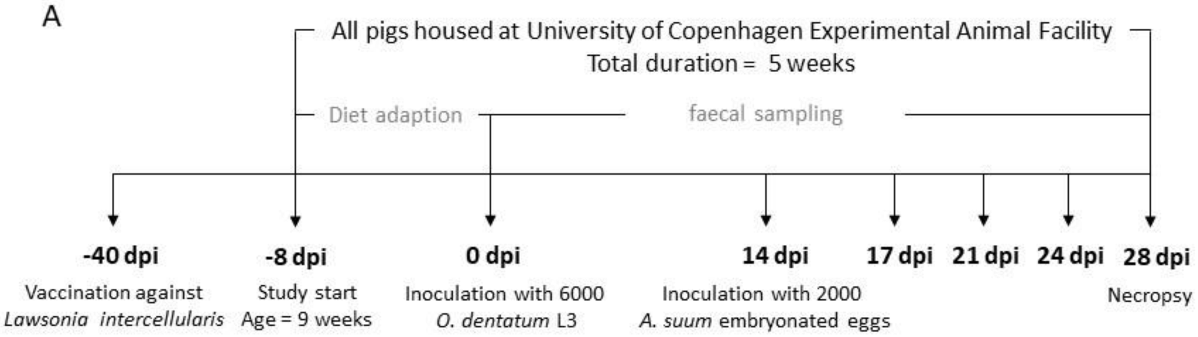

B

	**Control diet**	**Fer-SL diet** (8% fermented *S. latissima*)
**No infection**	UC<br>n = 8	USL<br>n = 8
***O. dentatum* and *A. suum* infection**	IC<br>n = 8	ISL<br>n = 8

Supplementary Figure 2 – Timeline and study design of Study 2. (**A**) 40 days prior to first infection, all pigs were vaccinated against *Lawsonia intercellularis*. At arrival (-8 dpi, age 9 weeks) 32 pigs were stratified according to weight and sex, and randomly allocated into four treatments groups (n=8). (**B**) Pigs were fed a control diet or a diet with 8% (w/w) fermented *Saccharina latissima* (Fer-SL) and half of the pigs fed each diet were infected with *Oesphagostomum dentatum* and *Ascaris suum*.

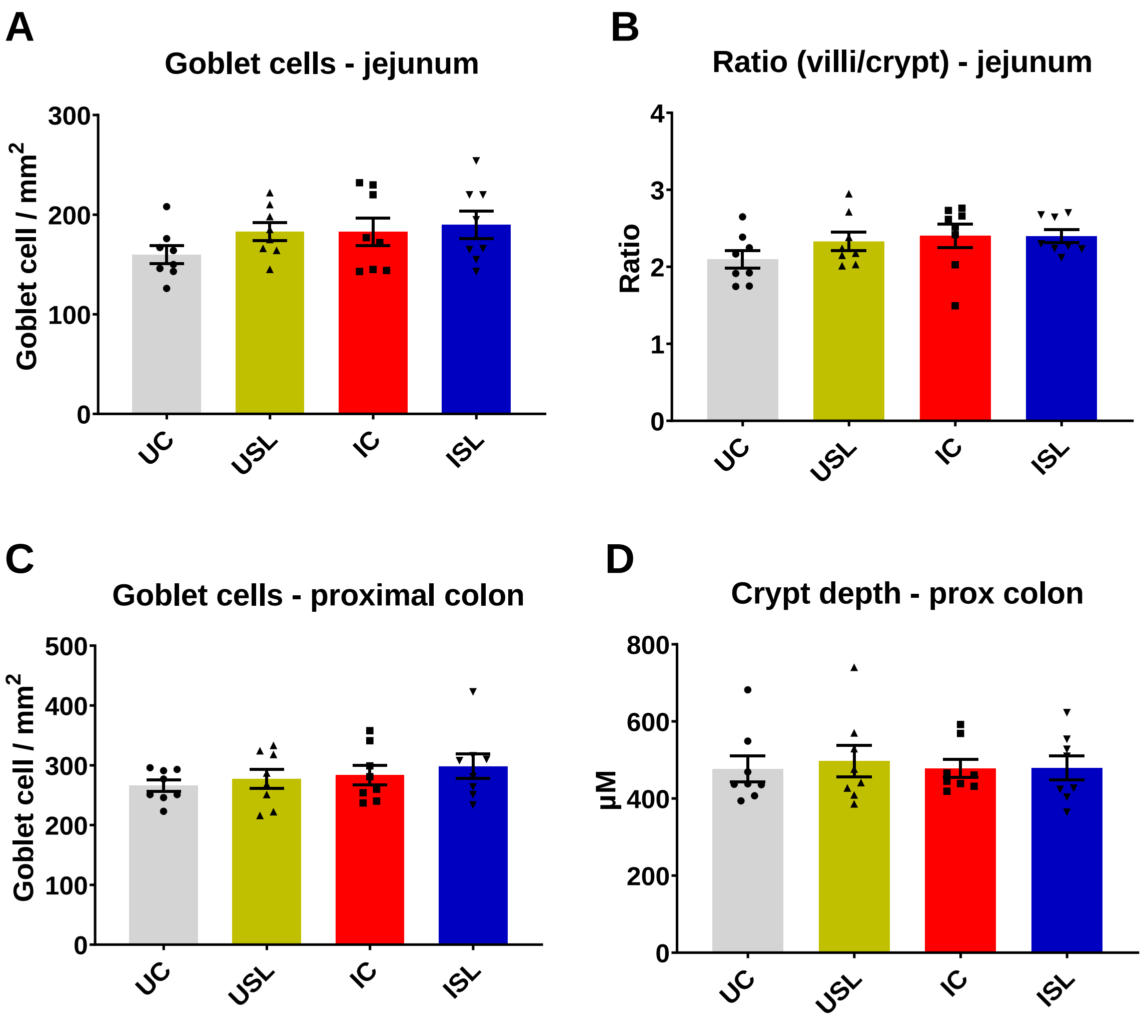

Supplementary Figure 3: (A) Goblet cells and (B) villi/crypt ratios of jejunum and (C) goblet cells and (D) crypt depth of proximal colon tissue from control group (UC), *Oesphagostomum dentatum* and *Ascaris suum* infection (IC), fermented *Saccharina latissima* supplementation (USL), or *O. dentatum* and *A. suum* infection combined with fermented *S. latissima* supplementation (ISL) (n=8). Data are presented as mean ± SEM with individual values.

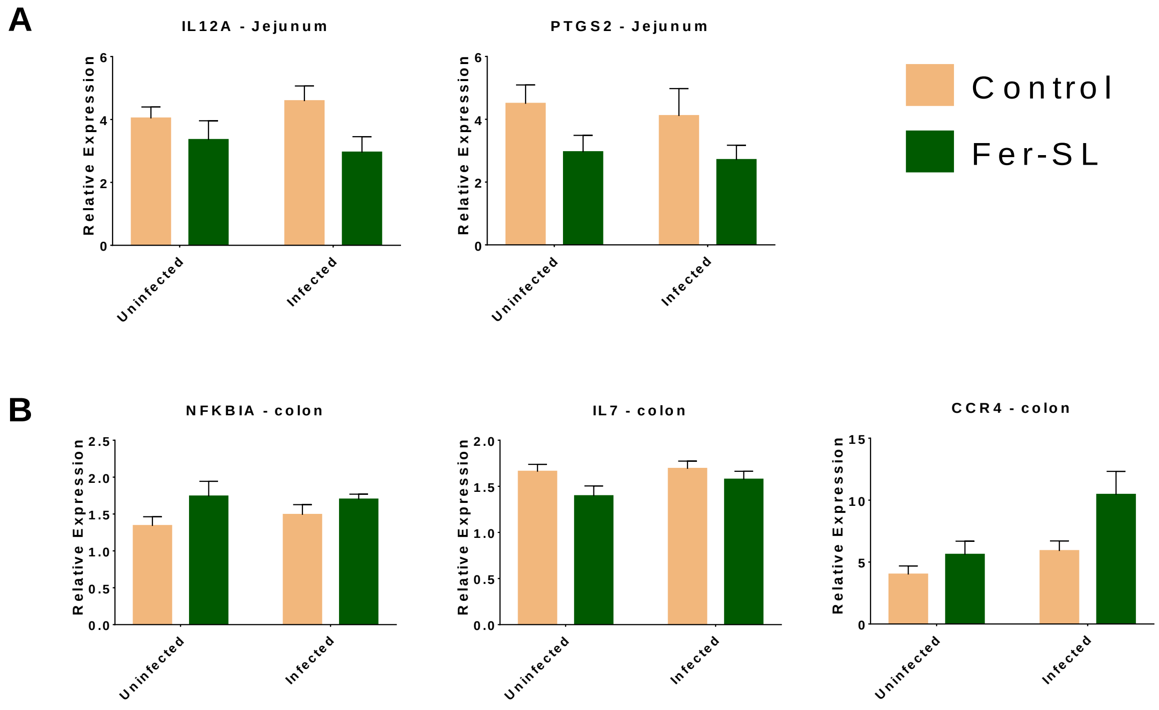

Supplementary Figure 4: Relative expression of significant genes in relation to diet (fermented *Saccharina latissima* (Fer-SL) or control diet) in groups of pigs (n=8) with or without parasite infection (*Oesphagostomum dentatum* and *Ascaris suum*) for (**A**) jejunum and (**B**) proximal colon (n=8). Figures illustrates mean ± SEM.

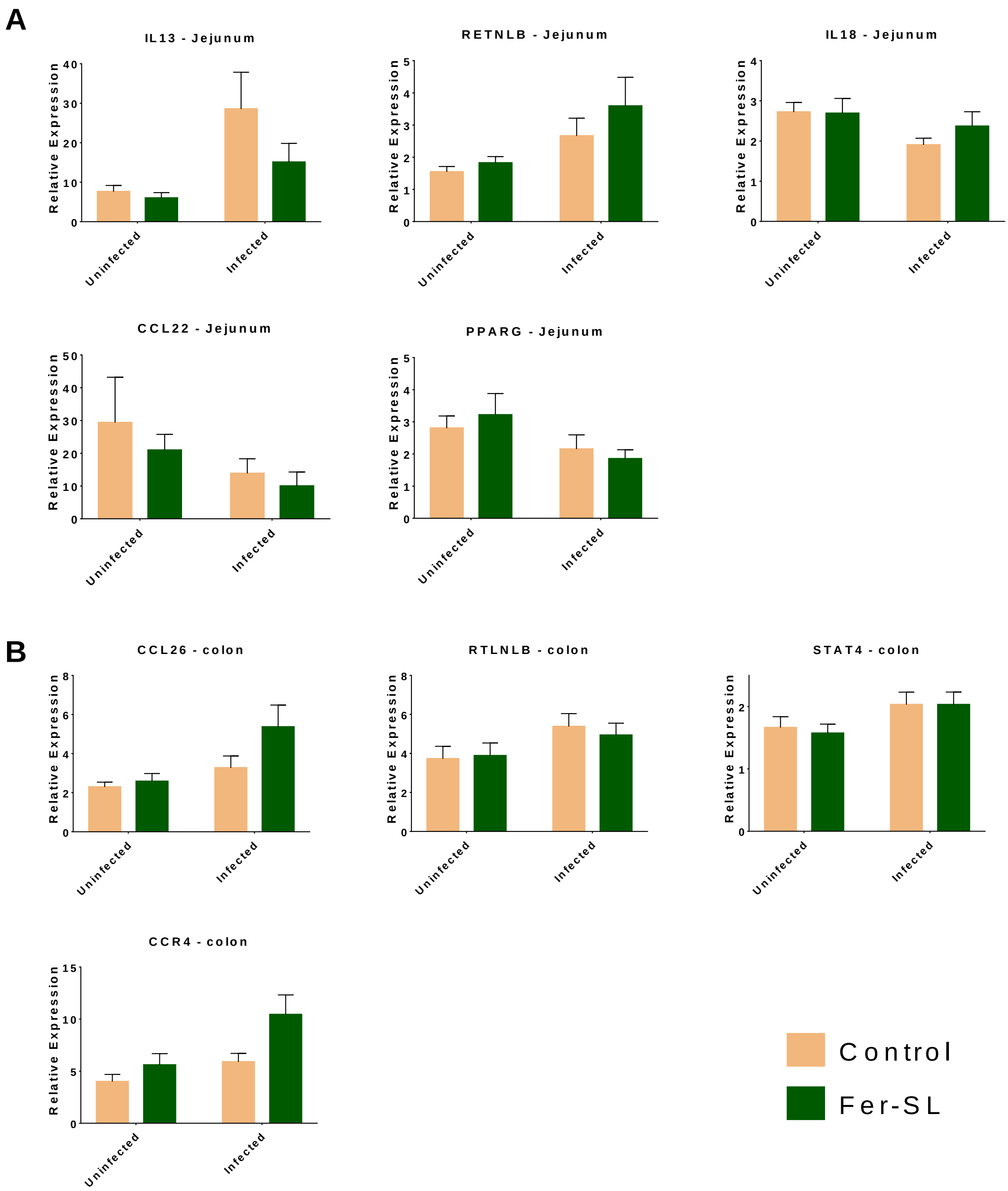

Supplementary Figure 5: Relative expression of significant genes on infection of parasite infection (*Oesphagostomum dentatum* and *Ascaris suum*) in groups of pigs (n=8) fed fermented *Saccharina latissima* (Fer-SL) or control diet for (**A**) jejunum and (**B**) proximal colon. Data are represented as mean ± SEM.

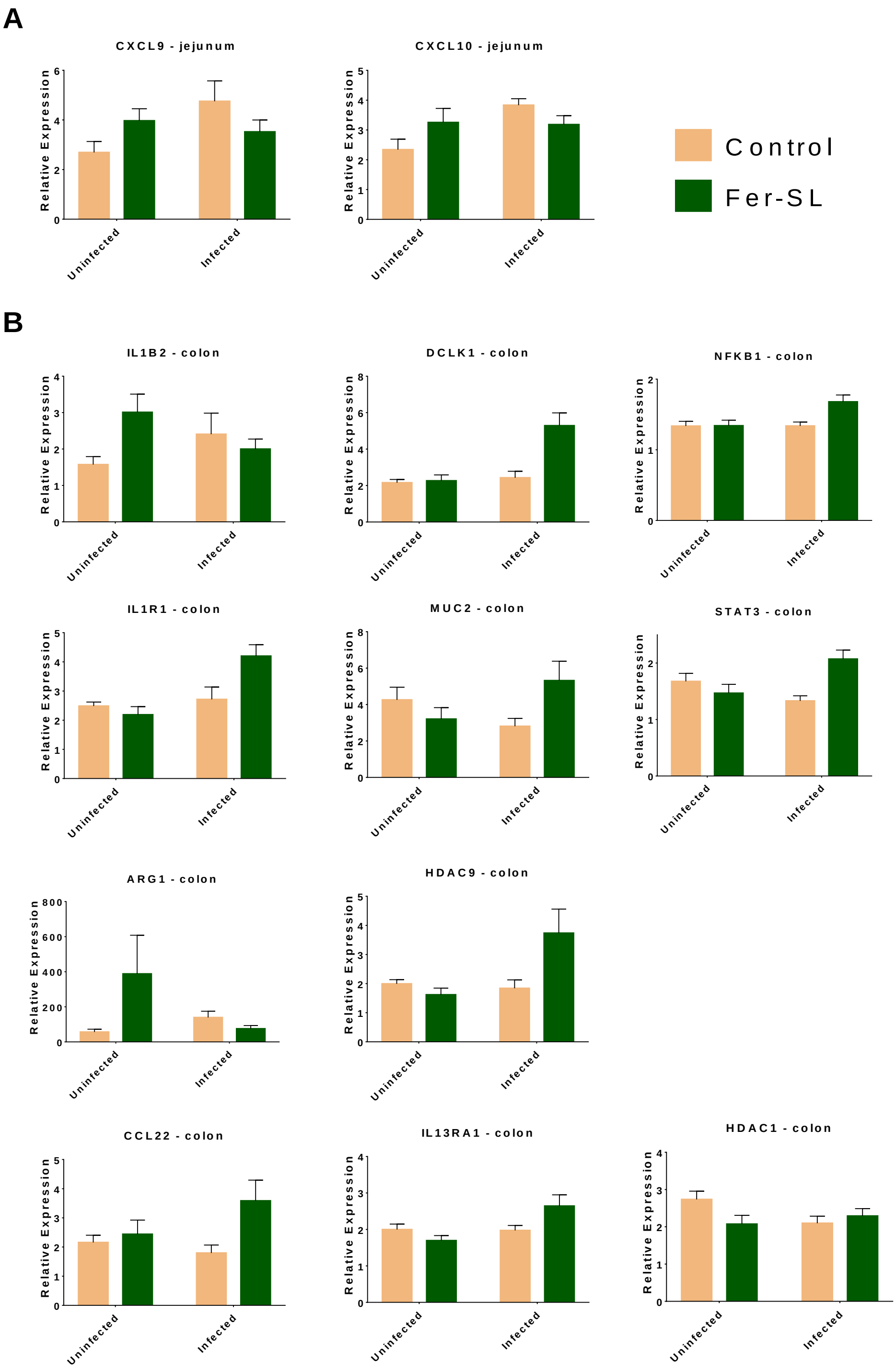

Supplementary Figure 6: Relative expression of significant genes in groups of pigs (n=8) on interaction between parasite infection (*Oesphagostomum dentatum and Ascaris suum*) and diet (fermented *Saccharina latissima* (Fer-SL)) for (**A**) jejunum and (**B**) proximal colon. Data are represented as mean ± SEM.

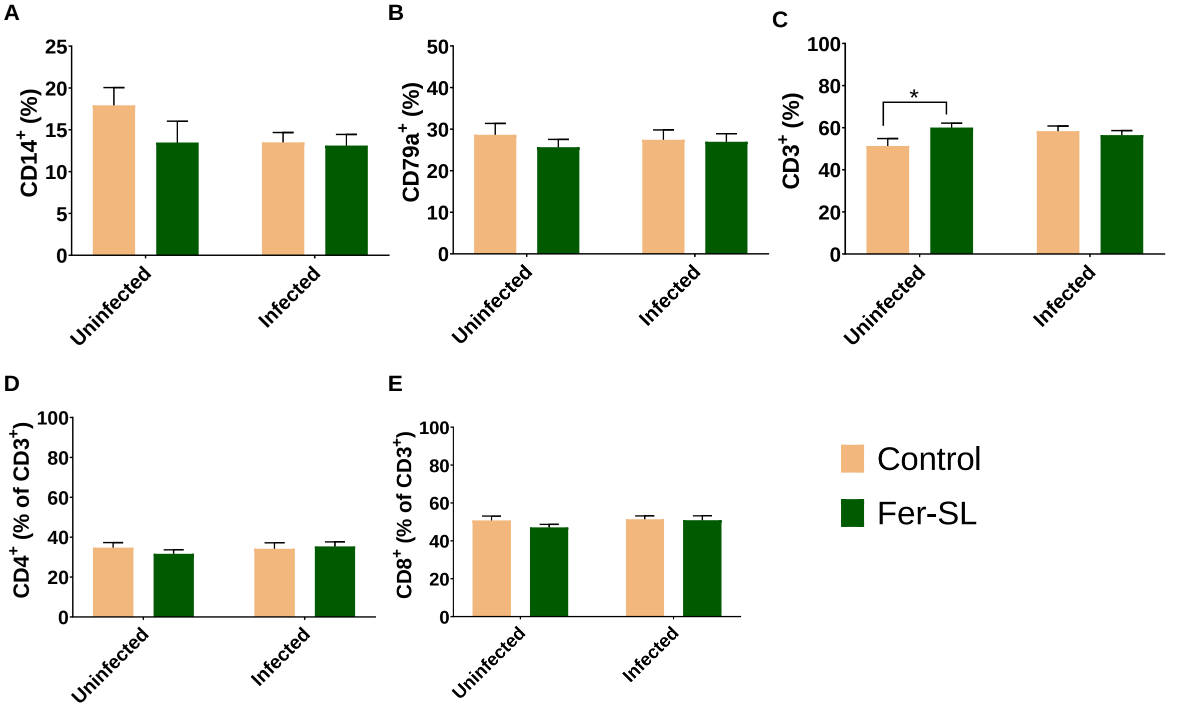

Supplementary Figure 7: Flow cytometric analysis was conducted on peripheral blood mononuclear cells (PBMCs) isolated from heparinized blood samples attained from all animals (28 dpi) in groups of pigs (n=8) infected with *Oesphagostomum dentatum* and *Ascaris suum* and fed either fermented *Saccharina latissima* (Fer-SL) or control diet. Data are presented as mean ± SEM for (**A**) monocytes (CD14+), (**B**) B-cells (CD79a+), (**C**) T-cells (CD3+), (**D**) T-helper cells (CD3+CD4+) and (**E**) T-cytotoxic cells (CD3+CD8+) (*P=0.05).

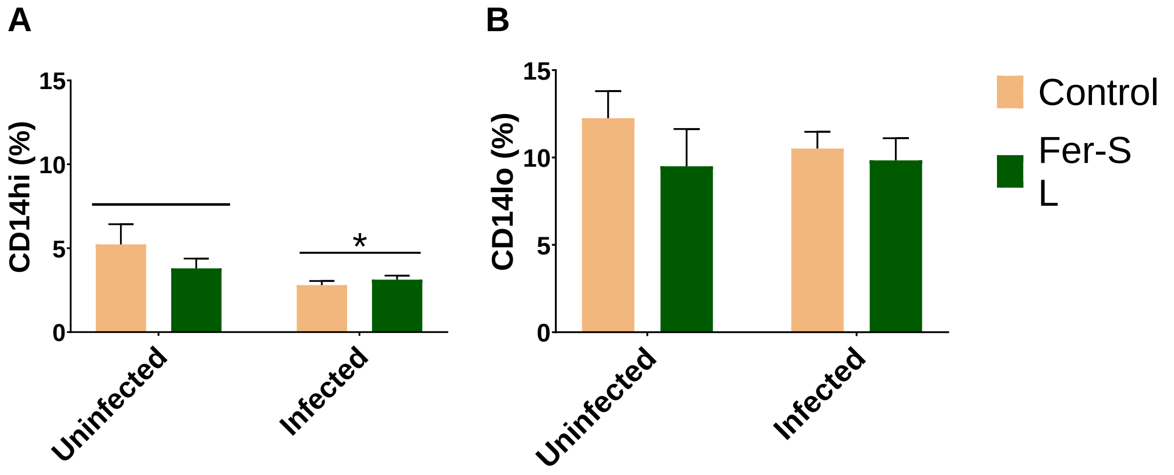

Supplementary Figure 8: Flow cytometric analysis was conducted on peripheral blood mononuclear cells (PBMCs) isolated from heparinized blood samples attained from all animals at necropsy (28 dpi) in groups of pigs (n=8) infected with *Oesphagostomum dentatum* and *Ascaris suum* and fed either fermented *Saccharina latissima* (Fer-SL) or control diet. Data are represented as mean ± SEM for (A) high avidity (CD14hi) and (B) low avidity (CD14lo) monocytes (*p ≤ 0.05).

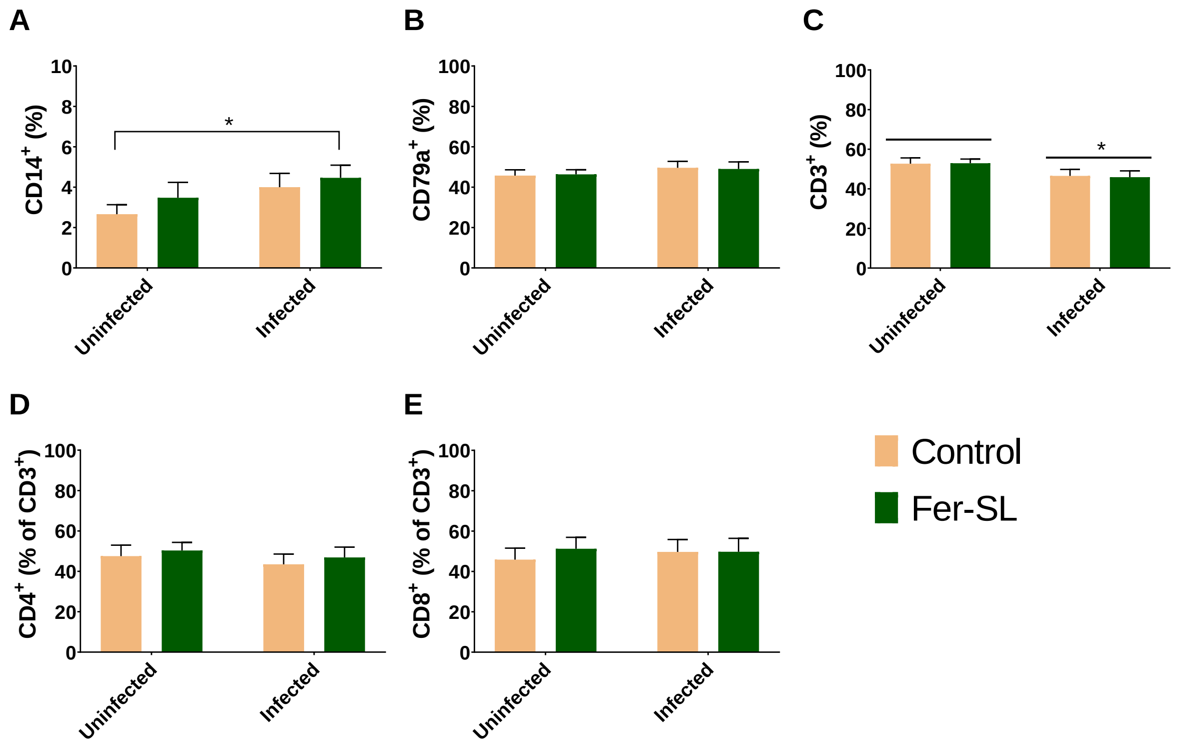

Supplementary Figure 9: Flow cytometric analysis was conducted on cells isolated from ileo-caecal lymph nodes (CLN) attained from all animals at necropsy (28 dpi) in groups of pigs (n=8) infected with *Oesphagostomum dentatum* and *Ascaris suum* and fed either fermented *Saccharina latissima* (Fer-SL) or control diet. Data are represented as mean ± SEM for (**A**) monocytes (CD14+), (**B**) B-cells (CD79a+), (**C**) T-cells (CD3+), (**D**) T-helper cells (CD3+CD4+) and (**E**) T-cytotoxic cells (CD3+CD8+) (*p ≤ 0.05) .
